# Mst4, a novel cardiac STRIPAK complex–associated kinase, regulates cardiomyocyte growth and survival and is upregulated in human cardiomyopathy

**DOI:** 10.1016/j.jbc.2024.107255

**Published:** 2024-04-03

**Authors:** Matthias Eden, Marius Leye, Justus Hahn, Emanuel Heilein, Marcin Luzarowski, Bill Völschow, Christin Tannert, Samuel Sossalla, Carlota Lucena-Porcel, Derk Frank, Norbert Frey

**Affiliations:** 1 Department of Internal Medicine III, University of Heidelberg, Heidelberg, Germany; 2 German Centre for Cardiovascular Research, Mannheim/Heidelberg, Germany; 3 Core Facility for Mass Spectrometry and Proteomics, Center for Molecular Biology at Heidelberg University (ZMBH), Heidelberg, Germany; 4 German Centre for Cardiovascular Research, Kiel, Lübeck, Hamburg, Germany; 5 Department of Cardiology, University Heart and Vascular Center Hamburg, Hamburg, Germany; 6 Department of Internal Medicine III (Cardiology and Angiology), University Hospital Schleswig-Holstein, Kiel, Germany; 7 Department of Cardiology, University of Giessen, Giessen and Kerckhoff Heart and Lung Centre, Giessen, Germany; 8 Tissue Bank of the National Center of Tumor Diseases (NCT) Heidelberg, Heidelberg University Hospital, Heidelberg, Germany

**Keywords:** cardiomyopathy, cardiac hypertrophy, phosphoproteomics, signal transduction, complex, heart failure

## Abstract

Myocardial failure is associated with adverse remodeling, including loss of cardiomyocytes, hypertrophy, and alterations in cell–cell contacts. Striatin-interacting phosphatase and kinase (STRIPAK) complexes and their mammalian STE20-like kinase 4 (Mst4) have been linked to development of different diseases. The role and targets of Mst4 in cardiomyocytes have not been investigated yet. Multitissue immunoblot experiments show highly enriched Mst4 expression in rodent hearts. Analyses of human biopsy samples from patients suffering from dilated cardiomyopathy revealed that Mst4 is upregulated (5- to 8-fold *p* < 0.001) compared with nonfailing controls. Increased abundance of Mst4 could also be detected in mouse models of cardiomyopathy. We confirmed that Mst4 interacts with STRIPAK components in neonatal rat ventricular cardiomyocytes, indicating that STRIPAK is present in the heart. Immunofluorescence stainings and molecular interaction studies revealed that Mst4 is localized to the intercalated disc and interacts with several intercalated disc proteins. Overexpression of Mst4 in cardiomyocytes results in hypertrophy compared with controls. In adult rat cardiomyocytes, Mst4 overexpression increases cellular and sarcomeric fractional shortening (*p* < 0.05), indicating enhanced contractility. Overexpression of Mst4 also inhibits apoptosis shown by reduction of cleaved caspase3 (−69%, *p* < 0.0001), caspase7 (−80%, *p* < 0.0001), and cleaved Parp1 (−27%, *p* < 0.001). To elucidate potential Mst4 targets, we performed phosphoproteomics analyses in neonatal rat cardiomyocytes after Mst4 overexpression and inhibition. The results revealed target candidates of Mst4 at the intercalated disc. We identified Mst4 as a novel cardiac kinase that is upregulated in cardiomyopathy-regulating cardiomyocyte growth and survival.

Despite significant recent therapeutic advances, heart failure remains a major cause of death worldwide. A main goal of current research therefore still is to analyze the fundamental molecules, molecular processes, and signaling mechanisms involved in order to better understand and therapeutically manipulate the interplay of maladaptive and protective signaling pathways (1, 2, 3, 4).

In the search for new potential candidate genes for heart failure and human cardiomyopathies, we recently discovered a previously uncharacterized protein, striatin-interacting protein 2 (Strip2), which we called "myocardium-enriched, calcium channel–associated protein" (Myoscape). Myoscape interacts with the cardiac L-type calcium channel (LTCC) and controls LTCC t-tubule localization and cardiac contractility (5). Strip2/Myoscape is a member of an evolutionary highly conserved complex termed striatin-interacting phosphatase and kinase complex (STRIPAK), an emerging signaling hub, so far largely uncharacterized within the heart. The “striatin family” consists of three proteins: striatin, the SG2 nuclear autoantigen (Sg2na/striatin3) and zinedin, which together act as core scaffolds within STRIPAK, binding other proteins *via* coiled coil domains (6, 7). STRIPAK includes an associated phosphatase, protein phosphatase 2A (Pp2a), which has been extensively studied in the heart and accounts for most cardiac dephosphorylation processes (8, 9, 10, 11). Typically, subunits A and C of Pp2a are part of STRIPAK complexes. For Pp2a, STRIPAK members appear to substitute for the regulatory subunit B (7). The main STRIPAK kinases are represented by the mammalian sterile 20-like kinases, Mst3 and Mst4 (12, 13, 14). The STRIPAK adaptor proteins Strip1 and Strip2/Myoscape are believed to bind striatins and Pp2a as well as Mst1/2 and Mst4, the latter *via* its C-terminal kinase binding domains (Δ421–744). In the case of Mst4, its kinase domain itself was determined to be solely responsible for binding the STRIPAK protein Slmap *via* its Forkhead-associated domain (15). Mst4 was initially described in 2001 and is regulated/activated by T-loop (auto)phosphorylation on T178 and binding of Mo25/cerebral cavernous malformations 3 (Ccm3) or the golgi protein GM130, to modify kinase activity and control localization. Mst4 as well as Mst3 are proteolytically cleaved by Caspase3 and form homodimers in multiple cell lines (16), whereas interaction with the STRIPAK-associated adaptor protein Ccm3 can recruit and thereby activate Mst3 to plasma membranes, less is known about upstream regulatory mechanisms of Mst4 (7). Besides localization to golgi and sarcolemma, Mst4 has been shown to localize and interact with cytoskeletal proteins, cell–cell junctions, the actinomyosin stress fibres. In respect to targets in noncardiac tissues, Mst4 is known to phosphorylate itself, Mst3, Acap4, Atg4b, Yap1, the ezrin, radixin, moesin component Ezrin and the MAP kinase Erk1/2 as well as the ubiquitin E3 ligase Traf6 (13, 16, 17, 18, 19, 20, 21, 22, 23). Moreover, in various cell types, a specific role for Mst4 in HIPPO pathway regulation and Pp2a antagonism has been shown, affecting cellular survival autophagy, tumor progression and metastasis (13, 16, 17, 18, 19, 20, 21, 22, 23).

In general, myocardial tissue can be regarded as functional syncytium composed of terminally differentiated myocytes that are electrically and mechanically coupled *via* highly organized and maturating cell–cell junctions known as intercalated discs (IDs) (24, 25, 26). Myocardial-IDs can be subdivided into gap junctions, desmosomes, the area composita, and adherens junctions with specialized molecular architectures and functions. As a crucial nodal point in mechanoperception and signal transduction, the molecular structure of IDs maturates throughout myocardial development and is differentially remodeled in disease and in response to hemodynamic stress (27). High wall stress increases the myocardial contractile force *per se* and also as a result of cardiac hypertrophy, and the ID structure adapts to this force (28, 29). In addition, human cardiomyopathies involving the right but also left ventricle have been associated with mutations in genes encoding structural and regulatory proteins of IDs, involved in mechanical coupling and signal transduction (3, 24, 28, 30). Constant, but also, rapid adaptation to changes in myocardial pressure load in physiological and pathophysiological conditions thus also require structural changes in the components at the ID level. These adaptation mechanisms include regulation of gene expression but also short-term available and adaptable mechanisms such as phosphorylation. Recently, phosphoproteomics studies in cardiomyopathy patient samples have also shown that proteins of the ID are target structures of high-consequence phosphorylation events (29, 31, 32).

## Results

### Mst4 is a component of STRIPAK complexes in cardiomyocytes

**Figure 1:**
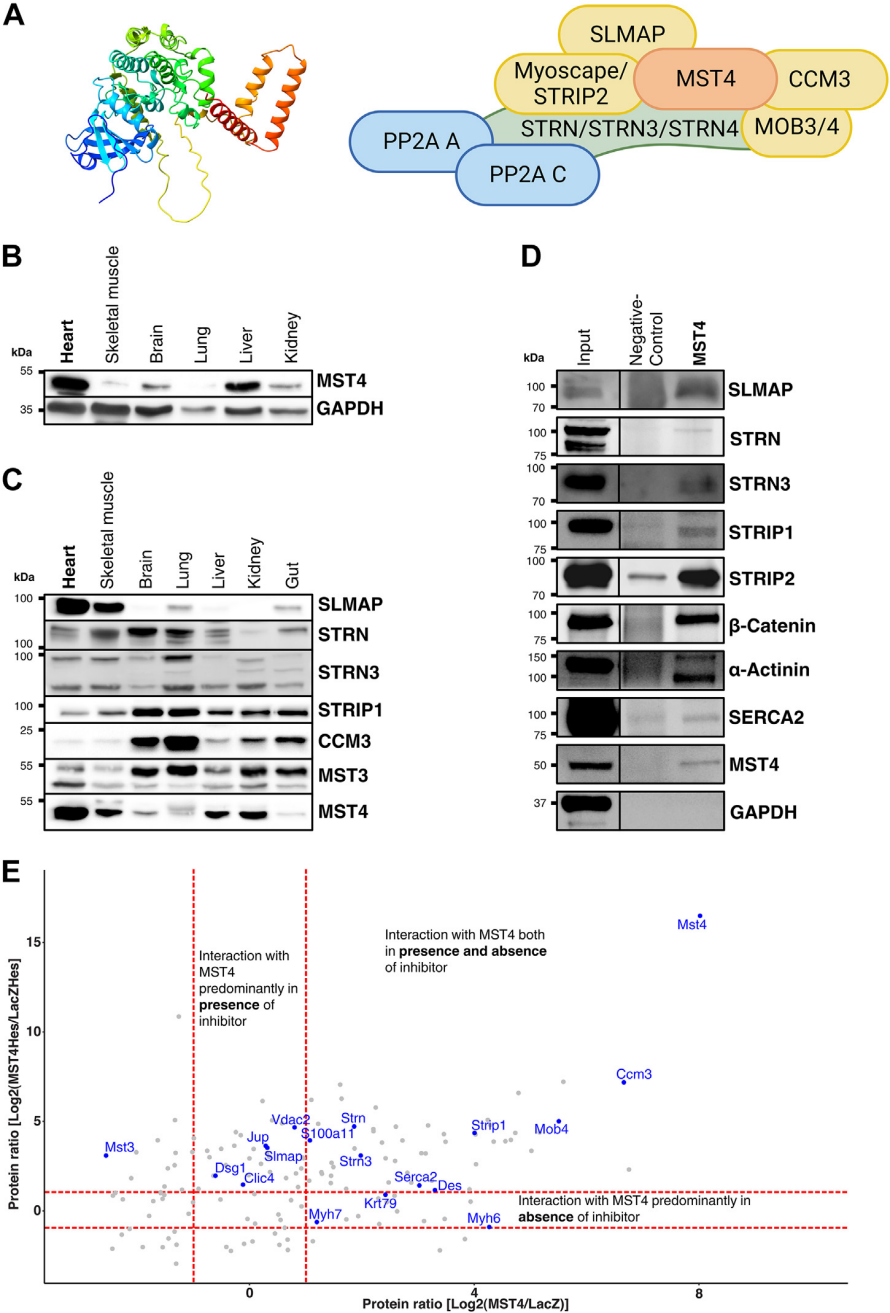
**Mst4 as a member of cardiac STRIPAK complexes.***A*, Mst4 predicted protein structure (*left*). *Right*: schematic model of the cardiac STRIPAK complex assembly. *B*, immunoblot results with protein lysates from different tissues of adult rat showing strong Mst4 expression in the heart. Gapdh expression serves as loading control. *C*, expression of various STRIPAK proteins in different tissues of adult mouse confirming high abundance of Mst4 in the heart as well as skeletal muscle, liver, and kidney. *D*, native coimmunoprecipitation in NRVCM searching for Mst4 protein interactors by fishing for Mst4. Immunoblotting using the respective antibodies confirmed an interaction of Mst4 with STRIPAK core proteins Slmap and striatin, striatin3, as well as Strip1 and Strip2/Myoscape. Moreover, Interaction of Mst4 protein with intercalated disc protein ß-Catenin, Z-disc, and intercalated disc protein α-actinin 2 and sarcoplasmic reticulum protein Serca2a is shown. *E*, interactome analysis and coimmunopreciptitation results in NRVCM in absence or presence of hesperadin and after LacZ or Mst4 overexpression were conducted using mass spectrometry. Results are displayed as *scatter plot* analysis with interaction depending on Mst4 overexpression (*x*-axis) or on hesperadin treatment (*y*-axis). Ccm3, cerebral cavernous malformations 3 protein; Clic4, chloride intracellular channel 4; Des, desmin; Dsg1, desmoglein 1; Jup, junction plakoglobin; Krt79, keratin 79; Mob4, Mob family member 4; Mst3, mammalian STE20-like kinase 3; Myh6, myosin heavy chain 6; Myh7, myosin heavy chain 7; myocardium-enriched, calcium channel–associated protein; NRVCM, neonatal rat ventricular cardiomyocyte; S100a11, S100 calcium-binding protein A11; Serca2, ATPase sarcoplasmic/endoplasmic reticulum Ca^2+^ transporting 2; Slmap, sarcolemma-associated protein; Strip1, striatin-interacting protein 1; STRIPAK, striatin-interacting phosphatase and kinase; Strn, striatin; Strn3, striatin3; Vdac2, voltage-dependant anion channel 2.

Mst4 rendering (AlphaFold EMBLS-EBI (33)) shows an N-terminal lobe containing the kinase domain (amino acids 1–297, red) and the a C-helix and a C-lobe containing the activation loop and phosphorylation sites as well as a dimerization domain (amino acids 347–416, blue). Mst4 also contains a linker region (amino acids 298–346 yellow) with a protein-binding motif (green) (Fig. 1*A* left). We previously identified the STRIPAK adaptor protein Strip2/Myoscape as a heart and muscle enriched protein interacting with the LTCC and actinin2. Now, we investigated whether Strip2/Myoscape also interacts with other STRIPAK members in cardiomyocytes and whether STRIPAK complexes are present in the heart. Figure 1*A* (right panel) shows a proposed model of the assembly of eukaryotic STRIPAK complexes, including its core members. Thus, we first analyzed protein expression of Mst4 expression in different rat organs (Fig. 1*B*) and of various STRIPAK members in multiple tissues of mice (Fig. 1*C*). These experiments also revealed that STRIPAK members Slmap and the kinase Mst4 are enriched in rodent cardiac and skeletal muscle. Next, we analyzed the formation of cardiac STRIPAK as a multiprotein complex in the heart. *Via* endogenous coimmunoprecipitation (Co-IP) in neonatal rat ventricular cardiomyocyte (NRVCM), we could detect a direct interaction of Mst4 with Slmap, striatin, striatin3, Strip1, and Strip2/Myoscape. Beyond that we could also detect an interaction of Mst4 with the ID protein beta-catenin, cardiac alpha actinin2, and Serca2a (Fig. 1*D*). Moreover, additional interaction studies were performed using the combination of Mst4 overexpression and inhibition *via* hesperadin, followed by Co-IP of Mst4 protein in NRVCM and mass spectrometry (MS)based quantification of coeluting proteins. Interactions were quantitatively compared and plotted against each other in the scatter plot. Interactions that preferentially occurred under Mst4 overexpression are shown in the right part of Figure 1*E* (positive on *x*-axis). In the other dimension of the figure (*y*-axis), interactions under hesperadin treatment are plotted against control. In this experimental setup, we confirmed Mst4's interaction with STRIPAK proteins, including Strip1, striatin, striatin3, Ccm3, and Mps one binder kinase activator-like 4, as well as with Serca2a and desmin within cardiomyocytes. Importantly, these interactions persisted regardless of the presence of the kinase inhibitor hesperadin. We also observed a number of proteins, which interact with Mst4, dependent on presence of hesperadin. Among others, we found that interaction of Mst4 with Slmap but also with desmoglein and junctional plakophilin is preferable after addition of a kinase inihibitor (middle, upper area). Interestingly, an interaction with Mst3 was mainly observed without overexpression of Mst4, which could be explained by the formation of heterodimers, which are less likely in the presence of overexpressed Mst4 proteins. An interaction with Mst4, especially without inhibiting kinase function, was observed for Myh6 and Myh7, among others. A complete list of the results can be found in the Supporting information. Taken together, these results not only confirm the presence of a cardiac STRIPAK complex they also indicate that Mst4 interacts with several proteins of the contractile apparatus, the ID structure, and the excitation-contraction machinery.

### Mst4 localizes to cardiac IDs

**Figure 2:**
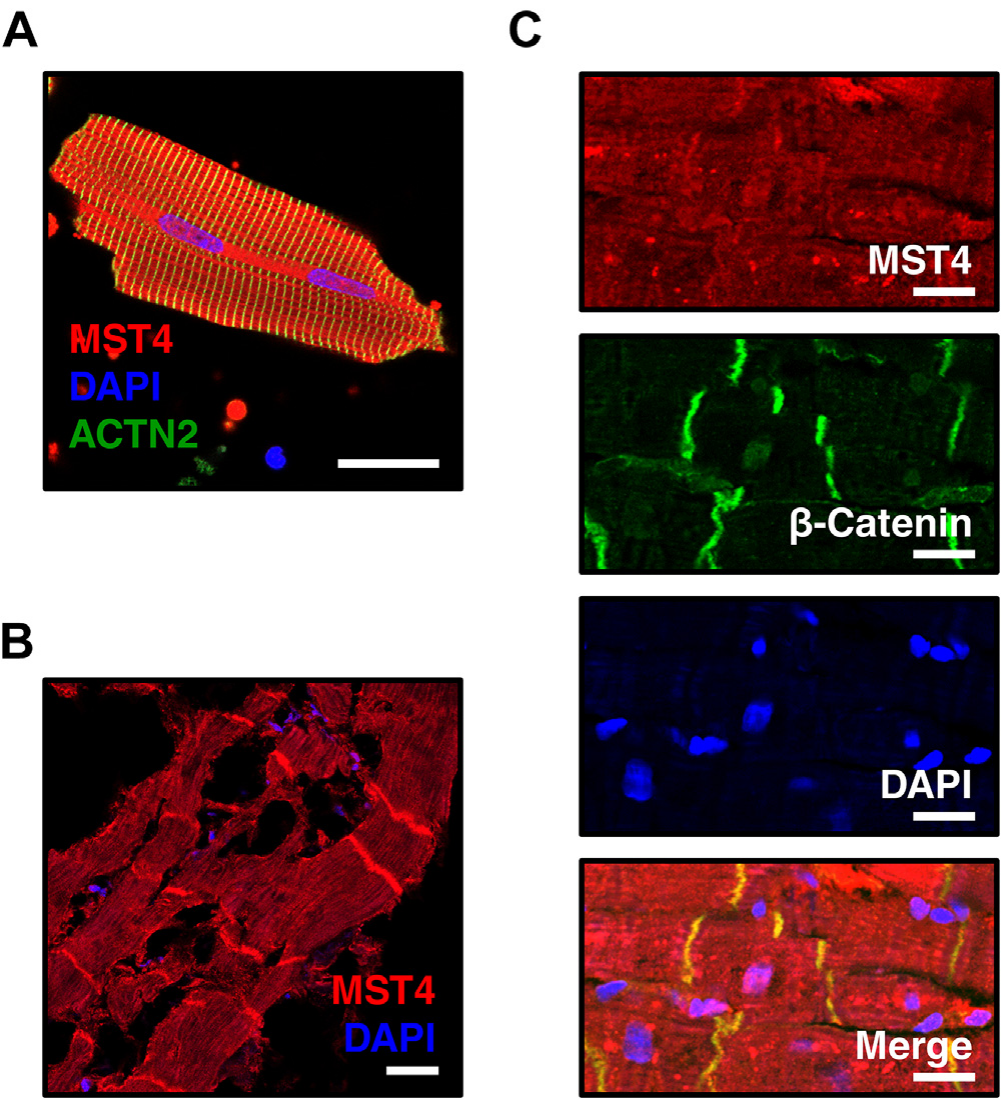
**Mst4 localization at the cell–cell junction in the heart.***A*, immunofluorescence stainings showing subcellular localization of Mst4 (*red*) in isolated adult rat cardiac myocytes costained with α-actinin (ACTN2, *green*) and in human cardiac tissue from DCM biopsy samples (*B*). The nucleus (*blue*) was stained with DAPI. *C*, immunofluorescence costainings of Mst4 protein (*red*) ß-catenin (*green*) showing Mst4 colocalization at the intercalated disc. The scale bars represent 20 μm. DAPI, 4´,6-diamidino-2-phenylindole; DCM, dilated cardiomyopathy; Mst, mammalian STE20-like kinase.

To assess the subcellular localization of Mst4 in human cardiac tissue and adult rat cardiac myocytes (ARVCMs), we next performed immunofluorescence experiments. Mst4 expression in isolated ARVCMs showed a predominantly longitudinal pattern with perinuclear enhancement (Fig. 2*A*). In human cardiac tissue from patients suffering from dilated cardiomyopathy, Mst4 staining showed a strong signal at cell–cell junctions (Fig. 1*B*). We performed additional staining in paraffin sections of ventricular myocardial biopsies from healthy transplanted human hearts (Fig. 2*C*). Here, we could show a colocalization of Mst4 with markers of the ID such as ß-catenin. Together with our interaction studies, this suggests a localization of Mst4 at the ID.

### Differential regulation of Mst4 under increased cellular densities and in human and experimental heart failure

**Figure 3:**
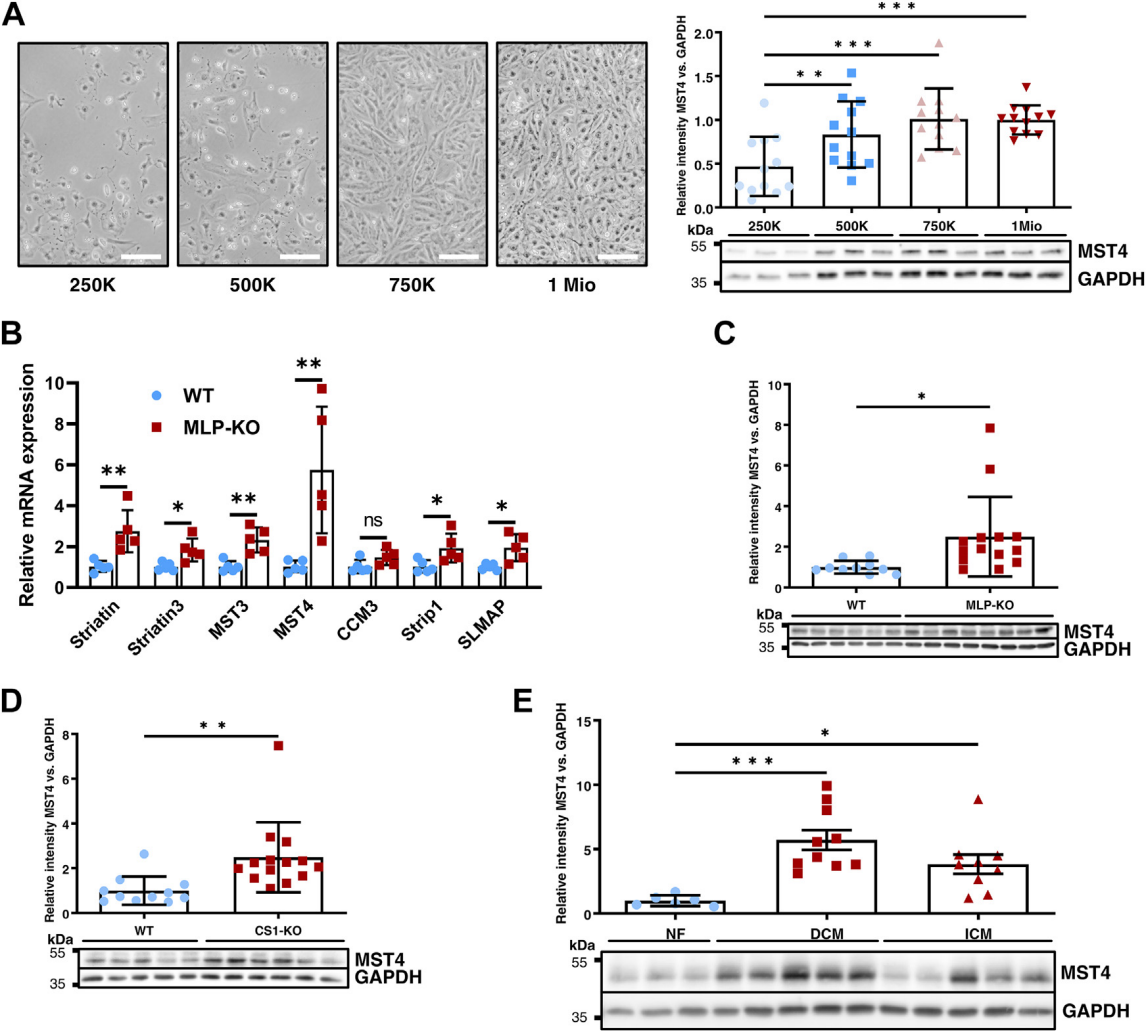
**MST4 differential regulation.***A*, Mst4 protein expression in cell culture conditions increases with increased NRVCM densities from 250,000 (ca. 26,000 per cm^2^) up to 1 million (ca. 104,000 per cm^2^) cells per well (example of culture plates on the *left*). Representative immunoblot results and statistical analysis from n = 3 experiments with three biological replicates each are shown on the *right*. (One-way ANOVA: *p* = 0.0003, F = 7.562). *B*, differential mRNA regulation of various STRIPAK members assessed *via* realtime quantitative polymerase chain reaction analysis in muscle LIM protein (MLP)-deficient mice (n = 5) and compared to WT littermates (n = 5). *C*, cardiac protein expression of Mst4 in MLP-KO mice (n = 14) compared to WT littermates (n = 10), representative immunoblot, and statistical analysis (*t* test: *p* = 0.0266). *D*, cardiac expression of Mst4 in calsarcin1 (CS1)-deficient mice (n = 14) compared to WT littermates (n = 11), representative immunoblot, and statistical analysis (*t* test: *p* = 0.007). *E*, Mst4 protein expression in human cardiac biopsy samples from patients either suffering from end stage dilated cardiomyopathy (DCM; n = 10) or ischemic cardiomyopathy (ICM; n = 9) also showed a significant upregulation of Mst4 protein compared to nonfailing controls (NF; n = 6). (one-way ANOVA: *p* = 0.0009, F = 9.775) (∗*p* < 0.05; ∗∗*p* < 0.01; and ∗∗∗*p* < 0.001). The scale bar represents 100 μm. MLP, muscle LIM protein; Mst, mammalian STE20-like kinase; NRVCM, neonatal rat ventricular cardiomyocyte; STRIPAK, striatin-interacting phosphatase and kinase.

**Figure S1:**
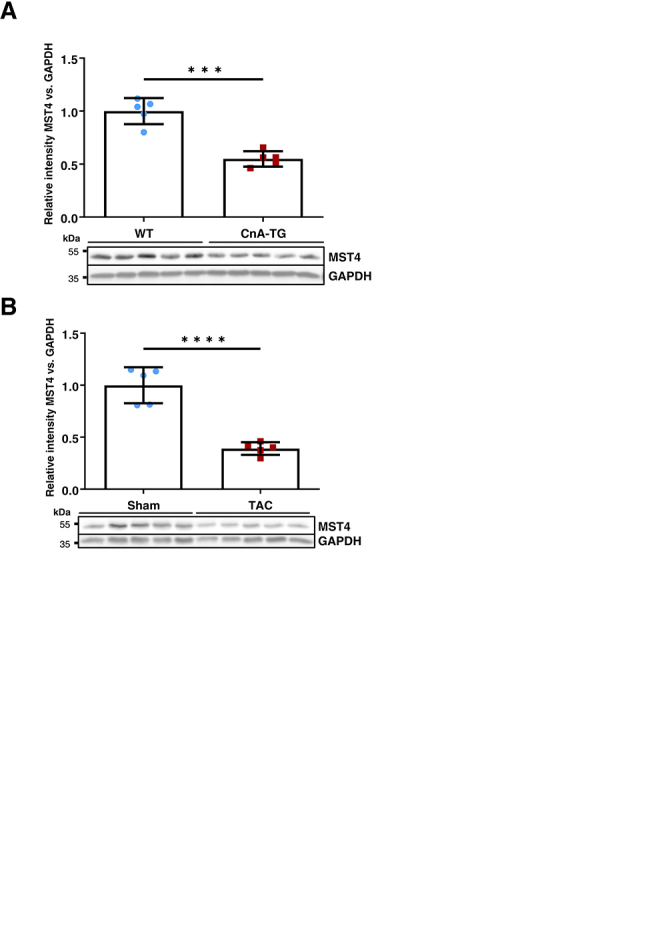
(A) Immunoblotting experiments comparing Mst4 protein abundance in mice with cardiac restricted transgenic expression of the pro-hypertrophic phosphatase calcineurin (CnA-TG; n = 5) compared to wildtype littermates (WT; n = 5) (*t* test: p = 0.0001). (B) Immunoblotting experiments comparing Mst4 protein abundance in mice after transverse aortic constriction (TAC; n = 5) compared to appropriate sham operated animals (n = 5) (*t* test: *p* < 0.0001). (∗∗∗*p* < 0.001; ∗∗∗∗*p* < 0.0001).

**Figure S2:**
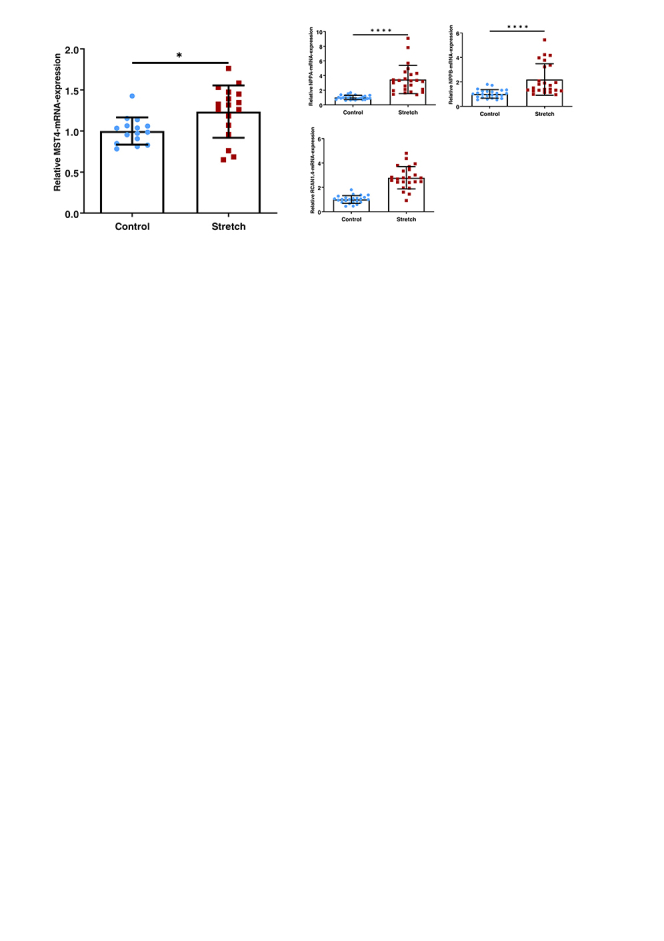
Increased MST4 (*t* test: p = 0.015) and also NPPA, NPPB, and RCAN 1.4 mRNA abundance (*t* tests: *p* < 0.0001) in NRVCMs subjected to bidirectional cyclic stretch (Flexcell systems) for 48 h and compared to nonstretched control conditions (n = 4 experiments with 3-6 biological replicates each); ∗*p* < 0.05; ∗∗∗∗*p* < 0.0001).

Since Mst4 protein is highly enriched in the heart, we analyzed its differential regulation in various cardiac stress models *in vitro* and *in vivo*. *In vitro*, we could detect strong Mst4 protein upregulation under increased cellular density in NRVCM culture (Fig. 3*A*). Strikingly, among the STRIPAK members analyzed, MST4 revealed the strongest upregulation on mRNA level in a rodent model of dilated cardiomyopathy (muscle LIM protein [MLP] KO mouse; Fig. 3*B*). In these MLP KO mice and also in calsarcin1 (CS1)-deficient mice, Mst4 protein expression was significantly increased (2.5-fold induction; *p* < 0.05 in MLP-KO and 2.5-fold induction; *p* < 0.01 in CS1-KO mice *versus* WT) (Fig. 3, *C* and *D*). Most importantly, Mst4 protein abundance was also significantly increased in myocardial samples from end stage heart failure patients with dilated cardiomyopathy (DCM) or ischemic cardiomyopathy compared to healthy, nonfailing control myocardium (Fig. 3*E*). In contrast, in mice with cardiac restricted overexpression of the prohypertrophic phosphatase calcineurin (CnA-transgenic), and in mice subjected to transverse aortic constriction, we observed a significant downregulation of Mst4 (Fig. S1, *A* and *B*). Cultured NRVCMs subjected to bidirectional cyclic stretch (FlexCell Systems) for 48 h showed increased MST4 mRNA abundance, while prohypertrophic markers like NPPA, NPPB, and RCAN1.4 gene expression were upregulated on mRNA level serving as a control (Fig. S2).

### MST4 enhances cardiomyocyte contractility *in vitro*

**Figure 4:**
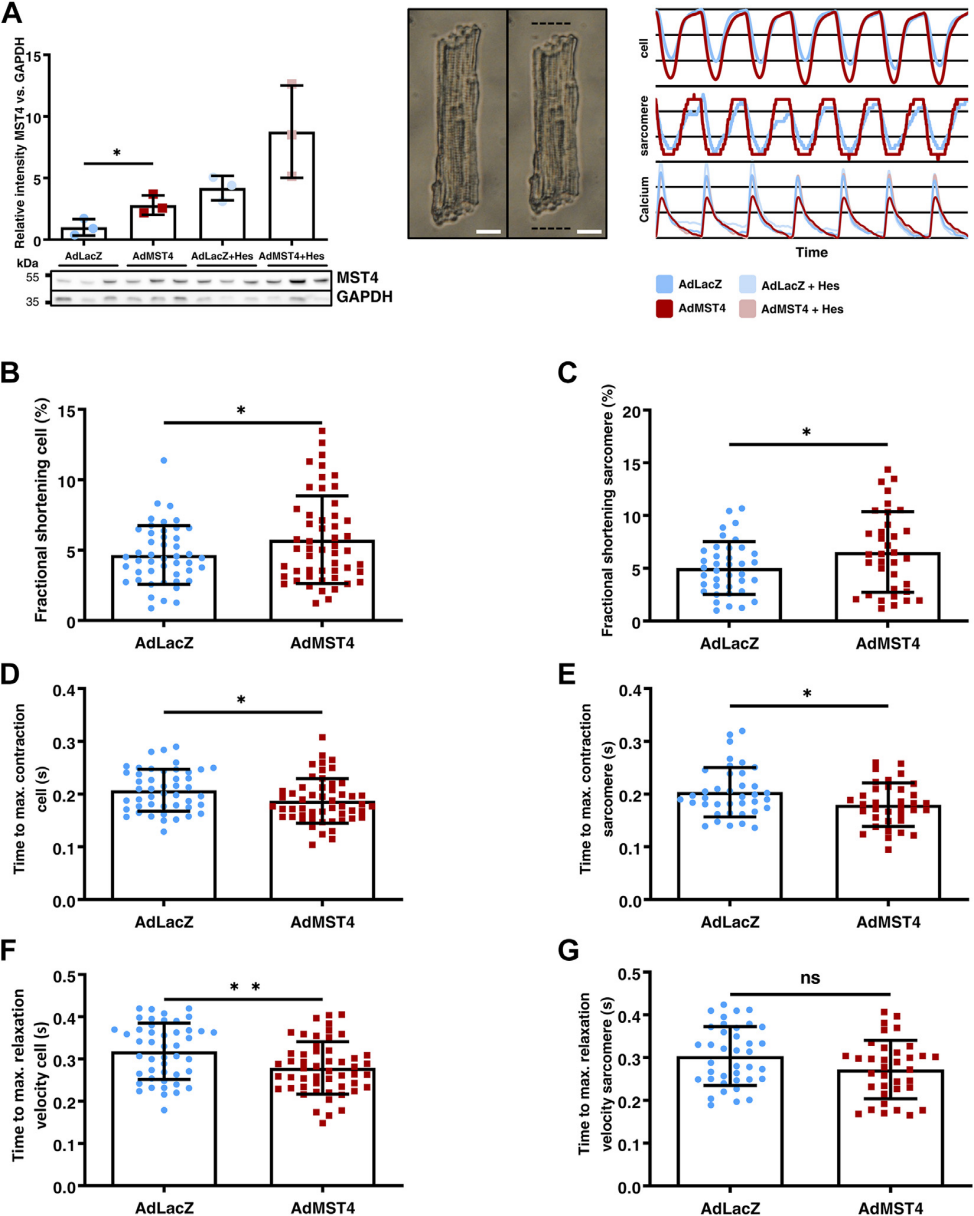
**MST4 overexpression results in enhanced cardiomyocyte contractility.***A*, contractility in adult rat ventricular myocytes (ARVCM) was assessed in the IonOptix calcium and contractility system after adenoviral Mst4 overexpression and compared to Lacz and also Mst4 overexpression and cotreatment with the kinase inhibitor hesperadin. Confirmation of significant protein expression and representative immunoblots are shown (*left*) (*t* test: *p* = 0.0387). ARVCM in relaxed (*middle left*) and contracted (*middle right, dashed lines* indicate relaxed length) state. Representative traces of relative cellular (*top*) and sarcomeric (*middle*) length as well as calcium concentration (*bottom*) are shown in the *right panel*. Cellular (*B*; *t* test: *p* = 0.0484) and sarcomeric (*C*; *t* test: *p* = 0.0465) fractional shortening, time to maximum contraction of cells (*D*; *t* test: *p* = 0.0182) and sarcomeres (*E*; *t* test: *p* = 0.0254), time to maximum relaxation velocity of cells (*F*; *t* test: *p* = 0.0029) and sarcomeres (*G*; *t* test: *p* = 0.0553) are displayed and were statistically significant improved after Mst4 overexpression. ∗*p* < 0.05 and ∗∗*p* < 0.01. The white scale bar represents 10 μm. Mst, mammalian STE20-like kinase; ns, not significant.

**Figure S3:**
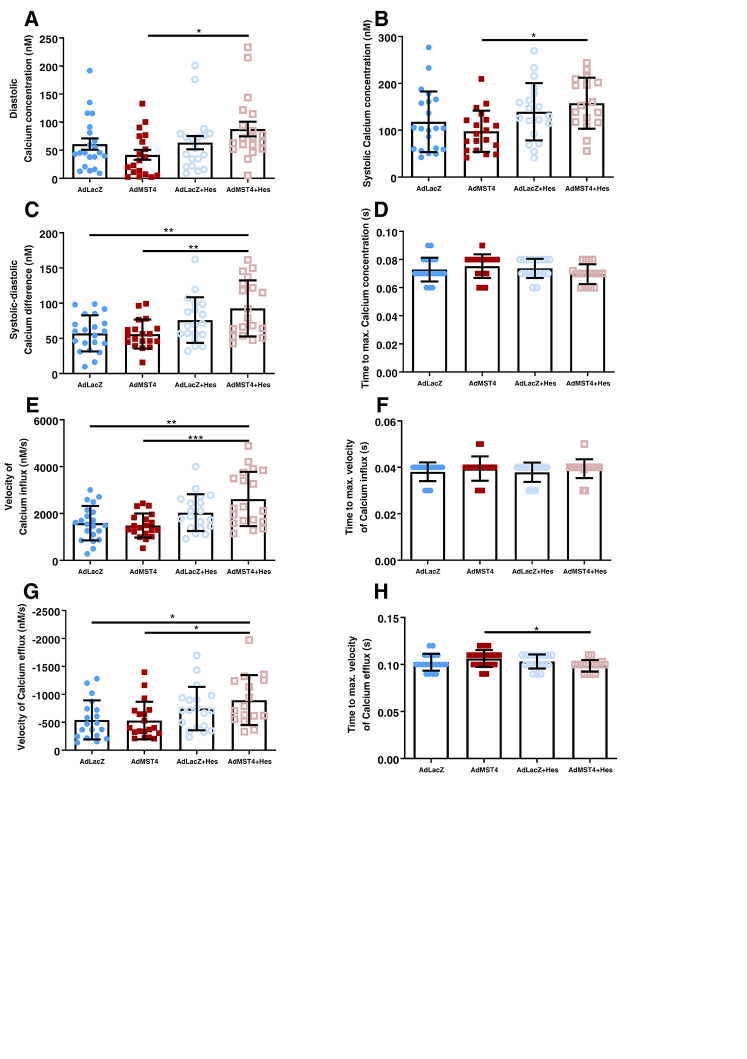
(*A*) Assessment of global calcium transients by FURA2-AM loading in ARVCM after Mst4 overexpression and co-incubation with hesperadin compared to LacZ and vehicle controls. Diastolic (*A*) systolic (*B*) and transients (*C*) are displayed. Also, the time to peak concentrations and the influx and efflux velocities of calcium transients were analyzed (*D*-*H*), showing significant impairment of calcium cycling after hesperadin treatment (∗*p* < 0.05; ∗∗*p* < 0.01).

As we previously identified the STRIPAK member Strip2/Myoscape as a novel regulator of cardiomyocyte calcium fluxes, we next asked whether Mst4 might also modulate cardiomyocyte calcium cycling and contractile function. Thus, we analyzed contractility and calcium cycling of ARVCMs using the IonOptix system and used adenoviral Mst4 overexpression in ARVCM and compared the results to LacZ as control. Moreover, we used Mst4 kinase inhibition with hesperadin treatment as an additional control. Of note, hesperadin treatment alone and in combination with adenoviral overexpression led to likely compensatory upregulation of Mst4 kinase (Fig. 4*A* left panel). Representative traces are shown on the right panel. Mst4 overexpression results in enhanced cardiomyocyte contractility and relaxation as shown by increased cellular and sarcomeric fractional shortening as well as reduced time to maximum contraction and time to maximum relaxation velocity (Fig. 4, *B*–*G*). Mst4 overexpression alone did not significantly alter global calcium transients in ARVCM, yet coincubation with its pharmacological inhibitor hesperadin significantly increased systolic and diastolic calcium contents as well as the velocity of calcium influx. Conversely, velocity of calcium efflux was reduced in cells overexpressing Mst4 and treated with hesperadin, potentially indicating involvement of other kinases blocked by hesperadin or counteraction of Mst4-dependent phosphorylation by cardiac phosphatases to stabilize calcium transients (Fig. S3, *A*–*H*).

### Mst4 promotes physiological cardiomyocyte hypertrophy

**Figure 5:**
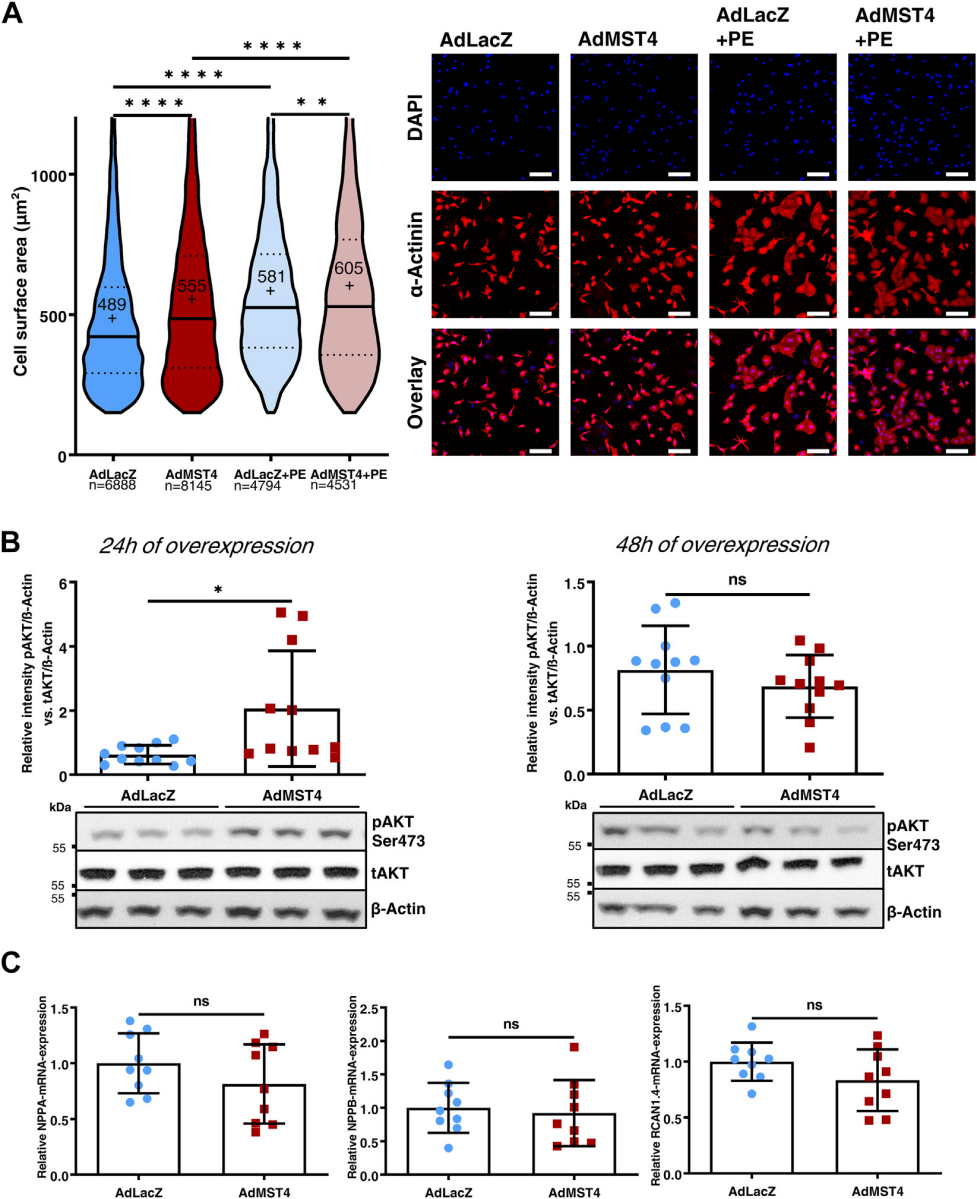
**Mst4 promotes physiological hypertrophy in neonatal rat cardiomyocytes.***A*, semiautomated cell size analysis of immunofluorescence images of NRVCM in which either LacZ (AdLacZ) or Mst4 (AdMST4) was adenovirally overexpressed and additionally treated with or without phenylephrine (+PE 100 μM). To only include NRVCMs, sarcomeric α-actinin and DAPI staining and detection was used. Statistical evaluation is displayed on the *left panel*. *Solid line* indicates median, *dotted lines* indicate quartiles, *cross* and *number* indicate the mean. Representative images are shown on the *right*. (n = 3 with three biological replicates each; one-way ANOVA: *p* < 0.0001, F = 152.8; the scale bar represents 100 μm). *B*, assessment of PKB (Akt) phosphorylation on serine 473 after 24 h of Mst4 overexpression (*left*; *t* test: *p* = 0.0167) and 48 h (*right*; *t* test: *p* = 0.3248) compared to LacZ as control (n = 3, three biological replicates each). Phosphorylation is displayed as ratio of phospho-Akt to total Akt normalized to beta-actin. *C*, qRT-PCR assessment of mRNA levels of NPPA (ANF; *t* test: *p* = 0.2273), NPPB (BNP; *t* test: *p* = 0.7073) and RCAN1.4 (*t* test: *p* = 0.1438) levels in NRVCM after MST4 overexpression for 24 h and compared to AdLacz (n = 3, three biological replicates each). ∗*p* < 0.05; ∗∗*p* < 0.01; and ∗∗∗∗*p* < 0.0001. DAPI, 4´,6-diamidino-2-phenylindole; Mst, mammalian STE20-like kinase; ns, not significant; qRT-PCR, quantitative real time PCR.

In order to investigate a potential effect of Mst4 on cardiomyocyte structure and growth, we performed semiautomated cell size analyses of immunofluorescence images of NRVCM in which either LacZ (AdLacZ) or Mst4 (adenovirus-encoding full-length Mst4 [AdMST4]) was adenovirally overexpressed and additionally treated with phenylephrine (+PE) or vehicle. To only include NRVCMs in the analysis, sarcomeric α-actinin and 4´,6-diamidino-2-phenylindole (DAPI) stainings were used. Semiautomated quantitative cell size assessment of several thousands of NRVCMs per group (n = 3 independent experiments) revealed that Mst4 overexpression resulted in significant but mild cardiomyocyte hypertrophy compared to LacZ overexpression. This effect could not be further increased by coincubation with prohypertrophic phenylephrine stimulation, though the overall differences remained rather small (ca. 100 μm^2^) (Fig. 5*A*; *p* < 0.0001). Of note, this hypertrophic phenotype induced by Mst4 overexpression was accompanied by an increase in phosphorylation of the protein kinase Akt on serine 473 (S473) when compared to controls (3.3-fold increase, *p* < 0.05, n = 3) (Fig. 5*B*) after 24 h of incubation. Akt activation in cardiomyocytes typically occurs in the context of “physiological” hypertrophy (34). Increased Akt phosphorylation was no longer significant after 48 h of Mst4 overexpression. Conversely, we could not detect increased expression of markers of pathological hypertrophy (“fetal gene program”) such as NPPA or NPPB, neither increased CnA activity (as assessed by RCAN1.4 expression), consistent with a phenotype of “physiological” hypertrophy due to Mst4 overexpression (Fig. 5*C*).

### Mst4 inhibits cardiomyocyte apoptosis *in vitro*

**Figure 6:**
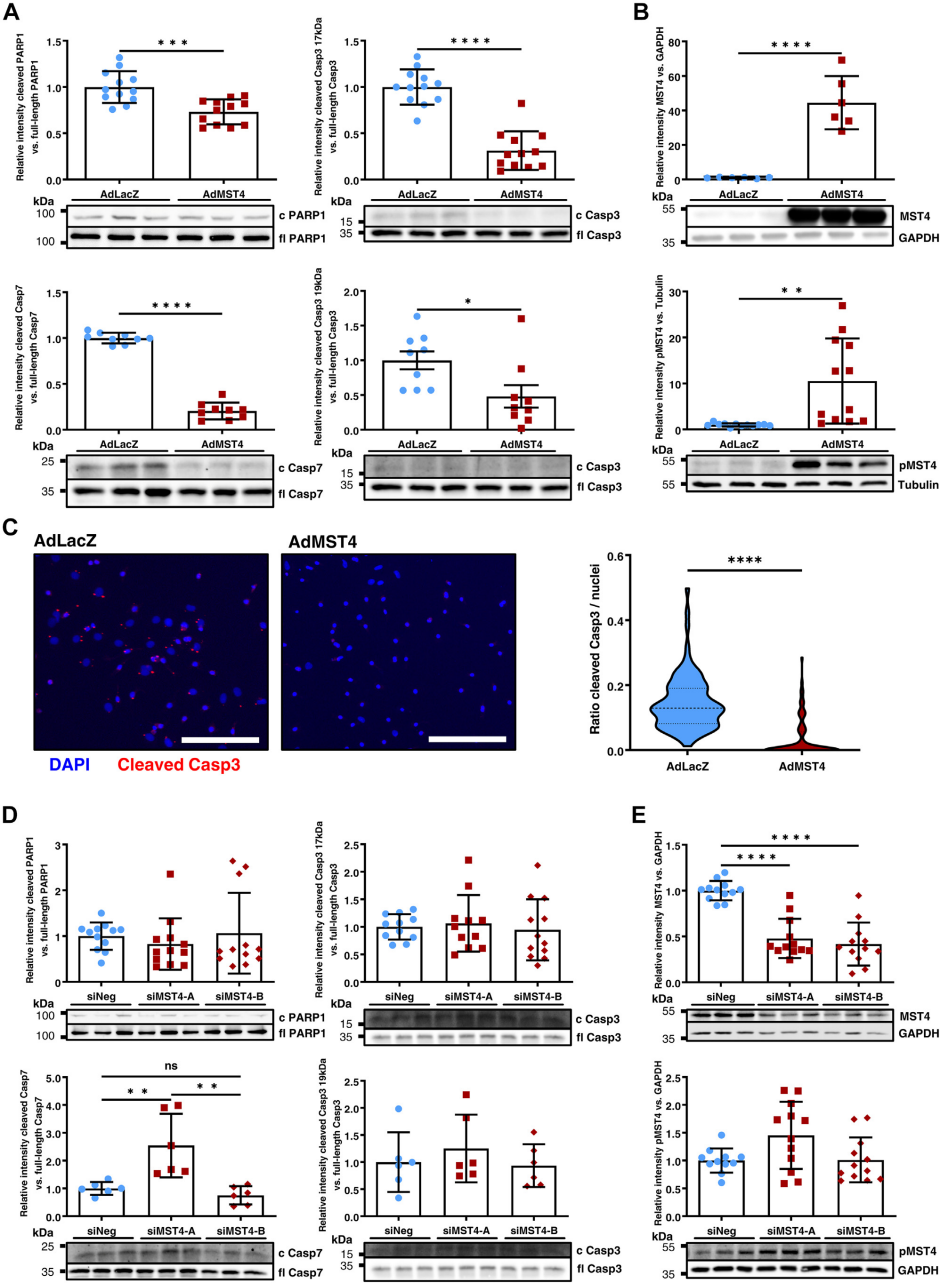
**MST4 inhibits apoptosis in neonatal rat cardiomyocytes.***A*, cleavage of Parp1 assessed by immunoblot (*top left panel*; *t* test: *p* = 0.0003), caspase7 (*bottom left*; *t* test: *p* < 0.0001), and caspase3 cleavage at 17 (*top right*; *t* test: *p* < 0.0001) or 19 kDA cleavage site (*bottom right*; *t* test: *p* = 0.0235) are shown after Mst4 overexpression compared to LacZ. (c Casp3 for cleaved product; fl Casp3 for “full length”). *B*, immunoblot of total Mst4 protein (*t* test: *p* < 0.0001) and Mst4 phosphorylated at T178 (pMST4; *t* test: *p* = 0.0017) after adenoviral Mst4 overexpression compared to LacZ, showing increased autophosphorylation of the kinase indicating increased activity. *C*, cleaved caspase3 cores (*red*) as assessed by immunofluorescence are shown. DAPI was used to stain cardiomyocytes nuclei (*blue*). Positive cores were assessed in relation to total DAPI positive nuclei. The scale bar represents 100 μm; *t* test: *p* < 0.0001. *D*, Mst4 downregulation by transfection of two different Mst4 mRNA-directed siRNA was achieved and resulted in increased caspase7 cleavage by siRNA A (one-way ANOVA: *p* = 0.0.0009, F = 11.51), whereas caspase3 (one-way ANOVA: 17 kD *p* = 0.831, F = 0.1862; 19 kD *p* = 0.5666, F = 0.5902) and Parp1 (one-way ANOVA: *p* = 0.6396, F = 0.4530) cleavage was unaffected. *E*, MST4 downregulation *via* siRNA transfection of NRVCM results in significant Mst4 protein knockdown with equal overall Mst4 phosphorylation at T178, indicating corresponding kinase activity. (∗*p* < 0.05; ∗∗*p* < 0.01; ∗∗∗*p* < 0.001; and ∗∗∗∗*p* < 0.0001). DAPI, 4´,6-diamidino-2-phenylindole; Mst, mammalian STE20-like kinase.

As the STRIPAK complex has been shown to be involved in cellular growth and survival in noncardiac tissues, we next investigated whether Mst4 is able to modulate cardiomyocyte apoptosis. Indeed, adenoviral overexpression of Mst4 in NRVCMs potently inhibited cardiomyocyte apoptosis, as shown by reduced cleaved Parp1 protein (Fig. 6*A* upper left panel) reduced cleaved Caspase7 (Fig. 6*A* bottom left panel), reduced cleaved Caspase3 protein at 19 or 17 kDa cleavage site (Fig. 6*A* right panel). Of note, we also detected an increased phosphorylation of Mst4 kinase by adenoviral overexpression at T178, which is associated with increased kinase activity (Fig. 6*B*) (16). In immunofluorescence studies, we observed reduced cleaved Caspase3 per nuclei as assessed by semiautomated analysis (Fig. 6*C*). In contrast, MST4 downregulation by transfection of Mst4 siRNA in NRVCMs (Fig. 6*D*) resulted in increased Caspase7 cleavage, whereas Caspase3 and Parp1 cleavage was unaffected. Interestingly, siRNA-mediated knockdown of Mst4 in NRVCM did not result in decreased phosphorylation of Mst4 on T178, indicating that the residual kinase protein was still active in knockdown and control conditions (Fig. 6*E*).

### Phosphoproteomics screen reveals potential targets for Mst4 in cardiomyocytes

**Figure 7:**
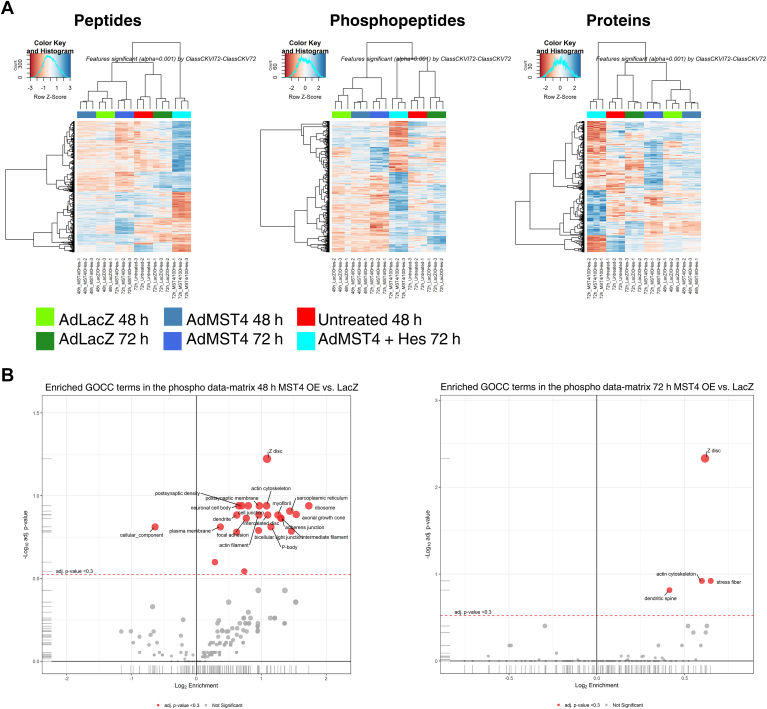
**Mst4 kinase targets in cardiomyocytes assessed by phosphoproteomics.***A*, cluster heat maps based on the significantly regulated peptides (*left*), phosphopeptides (*middle*), and proteins (*right*). The cluster heat maps based on the peptides, phosphopeptides, and proteins significantly regulated in contrast 4 (MST4 + Hes 72 h *versus* Mst4 72 h) show very well the effect of treatment of the respective experimental groups. Enriched GO terms after Mst4 overexpression for 48 h and 72 h compared to AdLacZ are shown in part (*B*). GO, gene ontology; Mst, mammalian STE20-like kinase.

Our findings revealed that Mst4 is a novel prohypertrophic, prosurvival kinase involved in cardiomyocyte hypertrophy, apoptosis, and contractility. Yet, its potential downstream phosphorylation targets that may mediate these effects are still unknown. Thus, we devised experiments to explore the phosphoproteome of Mst4. We utilized Mst4 overexpression at two time points and included conditions with kinase inhibition by hesperadin (35). Phosphoproteomics analyses were performed in neonatal rat ventricular cardiac myocytes. Bioinformatic and statistical analyses, including data integration and feature selection was applied at the peptide, phosphopeptide, and protein levels. Functional analyses of enriched terms, pathways, and enzyme substrates were also performed. Possible targets, which were significantly enriched by Mst4 overexpression compared to blank vector at the time points examined, and that could be significantly inhibited by hesperadin (indicating a true kinase target), were taken into account. These experiments revealed that Mst4 likely acts as a multitarget cardiac kinase, regulating and phosphorylating multiple proteins/peptides. Cluster heat maps based on the peptides, phosphopeptides, and proteins significantly regulated in different comparisons (AdMst4 + hesperadin *versus* AdMst4 without inhibitor compared to AdLacZ after 48 h and 72 h of treatment) show the effects of treatment of the respective experimental groups (Fig. 7*A*, regulated peptides left panel, regulated phosphopeptides middle section, and regulated proteins right panel). The replicates cluster closely together in each case, confirming the strong effect of each condition.

**Figure S4:**
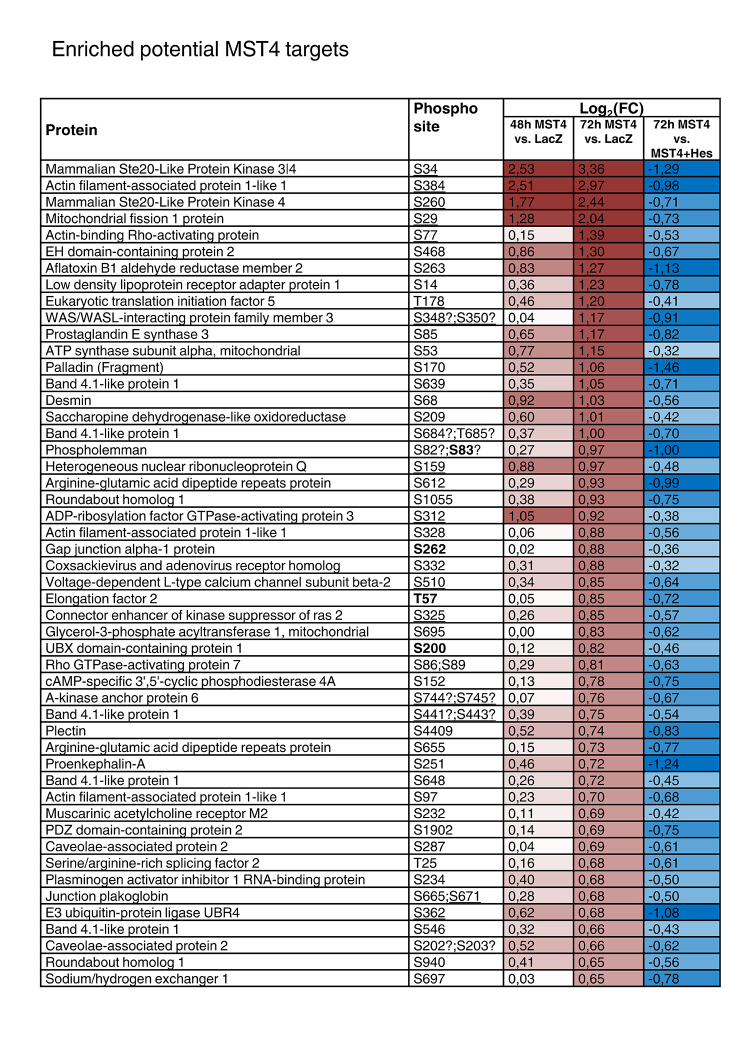
Table of selected enriched phosphoproteins and phosphorylation sites after 48 h and 72 h of Mst4 overexpression compared to LacZ and parallel hesperadin treatment (72 h AdMST4+hesperadin).

In total, we identified 74,721 peptide sequences, 20,973 phosphopeptides, and 4708 protein groups. These numbers are in the expected range for rat cell samples using SysQuant. 39,715 peptides, 8661 phosphopeptides, and 2753 proteins were quantified across all samples. We identified Mst4 peptides in all experimental groups with ∼70% sequence coverage. As expected, Mst4 abundance and levels of phosphorylation were substantially higher in NRVCM with adenoviral Mst4 overexpression. Comparison of proteomes in cardiomyocytes-overexpressing Mst4 ± inhibitor showed a stronger regulation both on the global peptide and phosphopeptide levels than the other groups, indicating that kinase inhibition with hesperadin still led to regulated phosphopeptides and regulated peptides. Interestingly, a phosphopeptide mapping to Mst4 (and shared with Mst3) containing pS34 was strongly downregulated after 72 h of hesperadin inhibited Mst4 overexpression compared to uninhibited Mst4 overexpression; whereas without the inhibitor, the same phosphopeptide showed an increase in kinase-overexpressing cells compared to blank vector. Surprisingly, the autophosphorylated site pT178 actually increased in abundance with inhibitor, and this may represent redundancy of phosphorylation by other kinases (which has been shown for PKA) or saturation of hesperadin in blocking complete kinase activity. Results of linear modeling on peptide and phosphopeptide level of the contrast 4 (Mst4 + hesperadin 72 h *versus* Mst4 72 h) showed that Mst4 + hesperadin at 72 h anticorrelates strongly to the groups Mst4 at 48 h and 72 h, consistent with a strong effect due to inhibitor treatment (Summary table in Fig. S4). Analysis for enriched gene ontology (GO) terms in the phosphodata matrix after 48 h or 72 h of Mst4 overexpression compared to AdLacZ showed enriched GO terms on the sarcomeric Z disc, actin cytoskeleton, stress fibres, and cell–cell junctions. Of note, after 48 h MST4 overexpression, GO terms of the sarcoplasmatic reticulum were also enriched (Fig. 7*B*).

**Figure S5:**
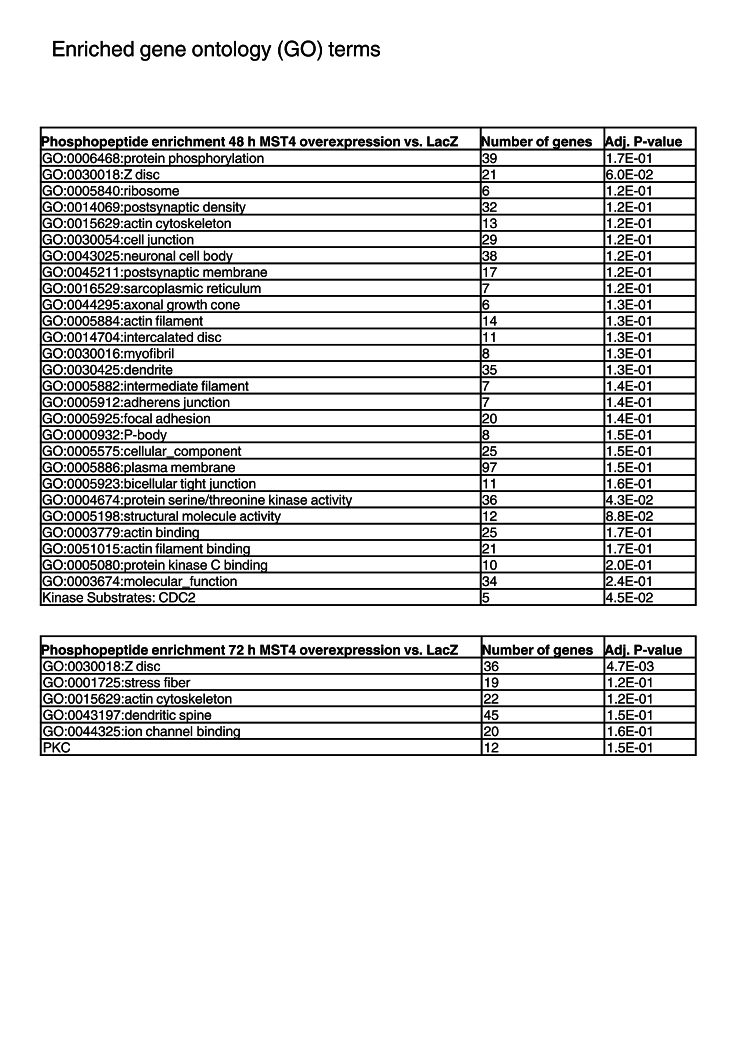
Tables showing enriched gene ontology (GO) terms after 48 h Mst4 overexpression (upper table) and 72 h Mst4 overexpression (bottom) compared to LacZ control.

As an additional proof of quality, we identified already published targets of Mst4 kinase in NRVCM, including protein phosphatase 1 regulatory subunit 14C at T72, between Mst3 and Mst4 shared autophosphorylation site T178, as well as a new autophosphorylation site on Mst3/4 at S34. New potential Mst4 kinase targets in cardiomyocytes included ryanodine receptor at S2684; Nfat5 at S635; phospholemman (Plm) at S83; Serca2A at S531 and S378; voltage-dependent LTCC subunit beta-2 at S692, S510, S214, and T215; Mlck2 at S422, S14, S15, and S113; gap junction alpha-1 protein at S325, T326, and S328; desmin at S68; junctophilin2 at T470, S479, and T483; myocardial zonula adherens protein at S18, junction Plakoglobin at S665 and S671; actin filament–associated protein 1–like one at S384; tight junction protein ZO-1 at S320; EF-hand domain–containing protein 2 at S468 as well as sodium/hydrogen exchanger 1 channel at S697 (Fig. S4). GO analysis of terms enriched for phosphopeptide abundance showed that possible targets and phosphopeptides found cluster in terms cell junction, ID, adherens junctions, focal adhesions, and bicellular tight junctions (Fig. S5), as well as sarcoplasmatic reticlulum and the muscular Z-disc.

**Figure S6:**
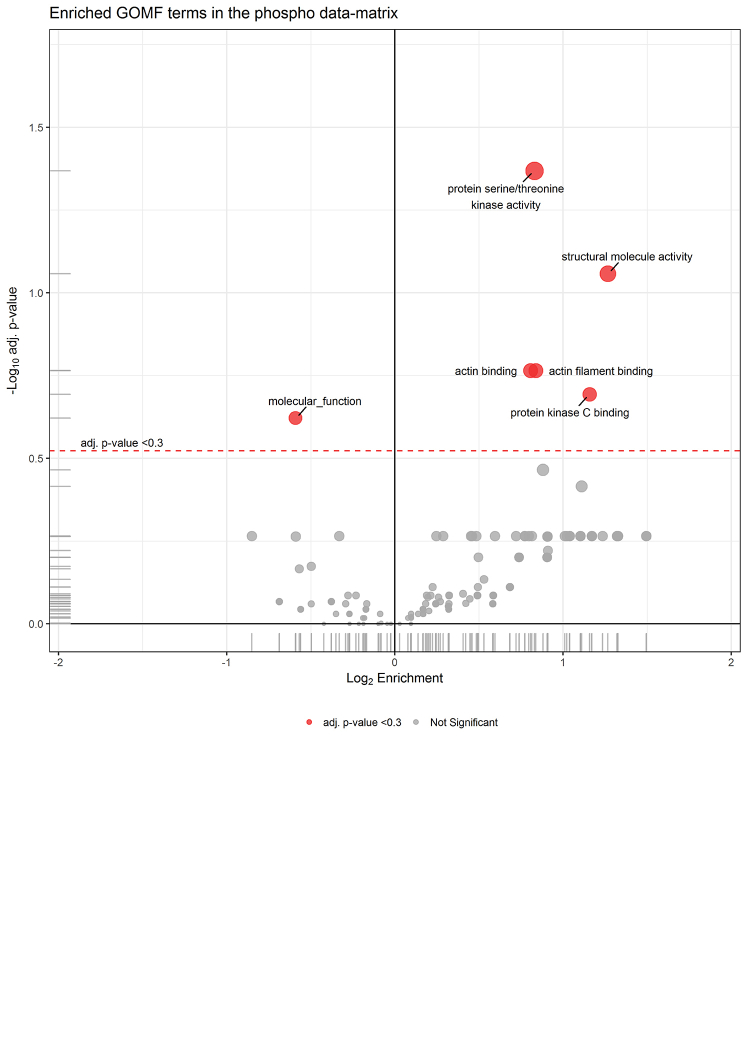
Phosphopeptide level GO molecular function (GOMF) enrichment for 48 h of Mst4 overexpression. Volcano plot showing log2 enrichment against significance of enrichment, highlighting the MFs found to be significantly over- (right side of zero) or under- (left of zero) represented by the differentially expressed peptides in the dataset. *Filled red circles* highlight significant hits (FDR<0.3) with the size related to the corresponding rank value.

**Figure S7:**
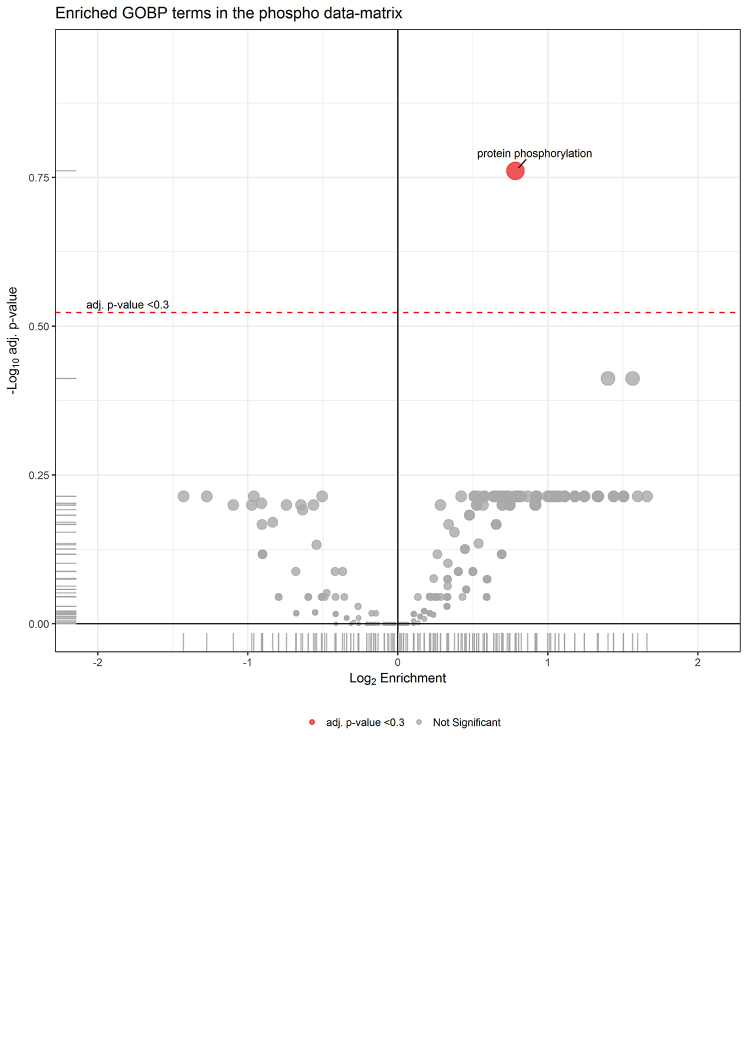
Phosphopeptide level GO biological process (GOBP) enrichment for 48 h of Mst4 overexpression. Volcano plot showing log2 enrichment against significance of enrichment, highlighting GO terms for biological processes found to be significantly over- (right side of zero) or under- (left of zero) represented by the differentially expressed peptides in the dataset. *Filled red circles* highlight significant hits (FDR<0.3) with the size related to the corresponding rank value. Protein phosphorylation was found to be highly significantly upregulated.

**Figure S8:**
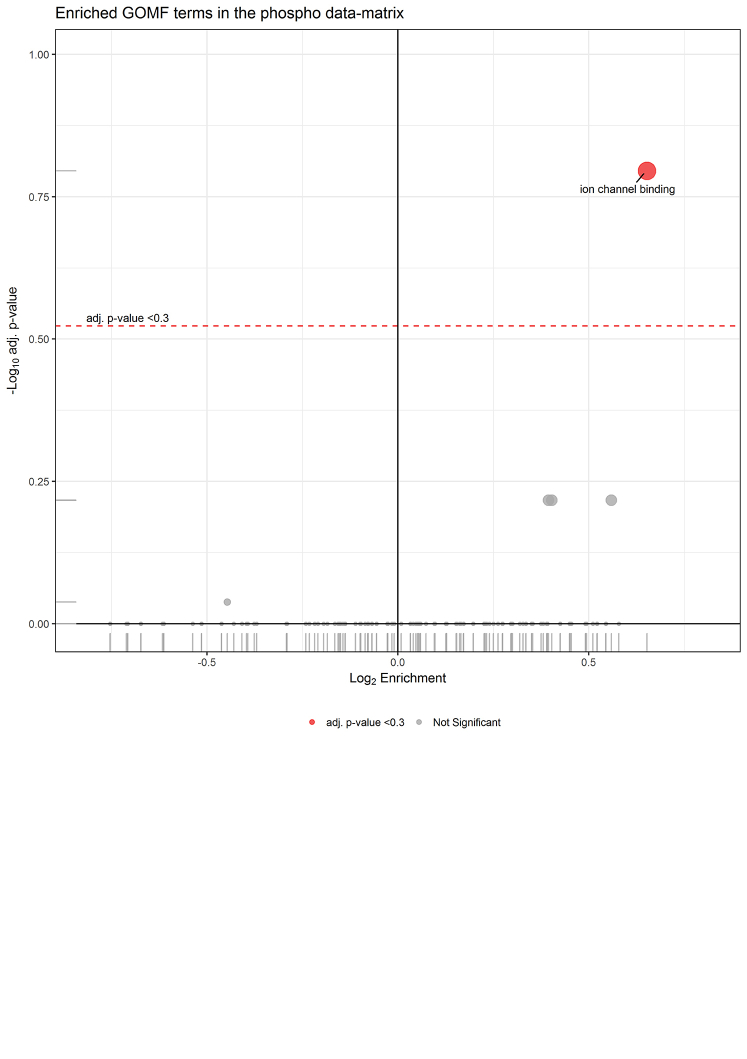
Phosphopeptide level GO molecular function (GOMF) enrichment for 72 h of Mst4 overexpression. Volcano plot showing log2 enrichment against significance of enrichment, highlighting GO terms for molecular functions found to be significantly over- (right side of zero) or under- (left of zero) represented by the differentially expressed peptides in the dataset. *Filled red circles* highlight significant hits (FDR<0.3) with the size related to the corresponding rank value.

**Figure S9:**
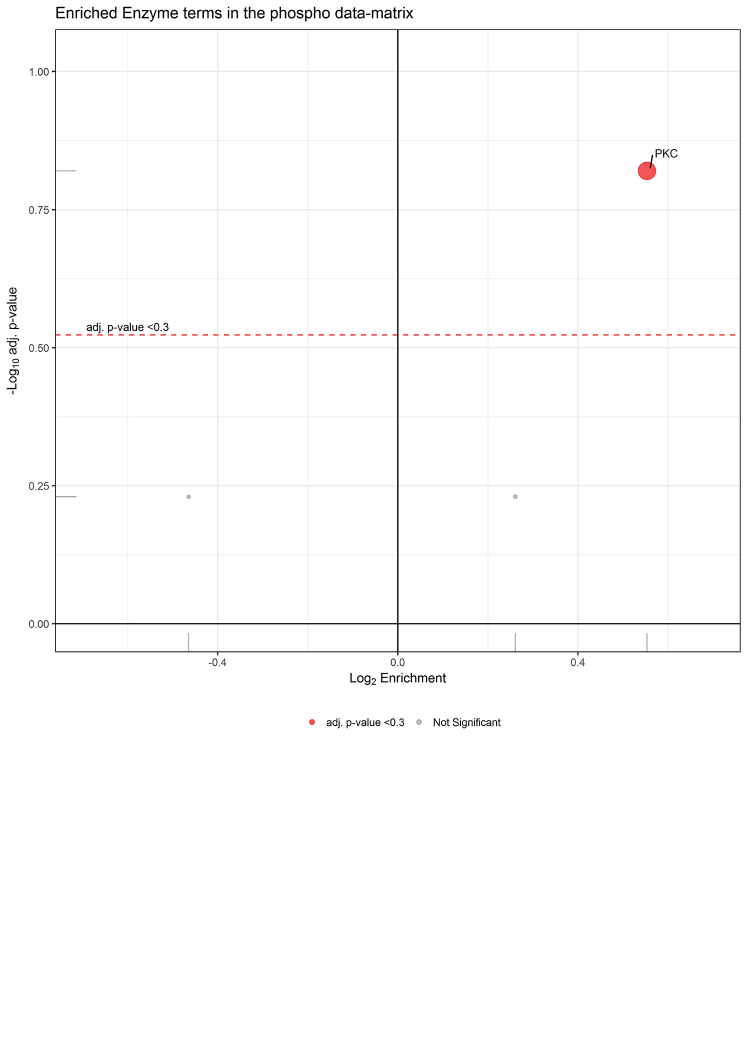
Phosphopeptide level enzyme terms enrichment for 72 h of Mst4 overexpression. Volcano plot showing log2 enrichment against significance of enrichment, highlighting enriched enzyme terms found to be significantly over- (right side of zero) or under- (left of zero) represented by the differentially expressed peptides in the dataset. *Filled red circles* highlight significant hits (FDR<0.3) with the size related to the corresponding rank value. Known Protein kinase C (PKC) phospho sites were highly significantly enriched upon Mst4 overexpression.

Together, these differentially phosphorylated targets analyzed in further GO term analysis highlight Mst4′s possible role in actin/stress fibre organization, Z-disc structure/function, processes at the IDs and ion/calcium channel function as well as cardiomyocyte contractility (Figs. S5-S9) and are consistent with the observed effects of Mst4 overexpression on cardiomyocytes.

## Discussion

A precise counterbalance of phosphorylation and dephosphorylation controlled and adapted by various kinases and phosphatases is crucial to maintain proper cardiomyocyte function (9, 11, 14, 30). Tightly regulated and correctly located protein kinases are indispensable to control cellular homeostasis not only in the heart but also in all organ tissues and thus represent potential targets for pharmacological interventions. The mammalian STRIPAK complex is a large multiprotein complex that is highly conserved among species and contains Pp2a subunits A and C as well as typically one or more sterile-20 kinases such as Mst3 and Mst4 (7). Additional core components of the mammalian STRIPAK complex are mainly striatin family protein members, Mps one binder kinase activator-like 3 and 4, Ccm3 (Pdcd10/Ccm3), and the Strip1 and Strip2 (Strip2/Myoscape). Beyond these core components, more than 100 variations have been described or predicted in various tissues, including additional adaptor proteins like Slmap, Cttnbp2/Cttnbp2nl, Traf3ip3, Sike1, and Caveolins (6, 7, 15, 36, 37).

STRIPAK members have also been linked to the ID in the past. Striatins in particular have been repeatedly associated with this structure and also heart diseases associated with the ID. Specifically, striatins are believed to provide mechanical stability to the myocardium *via* its expression at the ID (38, 39, 40), and its interaction with various desmosomal proteins like occludin and cingulin (41). The importance of striatins for ID function is further highlighted by studies, indicating that silencing the STRN gene reduced the expression of other ID proteins (ZO-1, E-cadherin, and occludin) (41). Consistently, the loss of striatin from the intercalated discs, along with the appearance of compromised desmosomal structures, were documented in cardiovascular disorders namely in patients suffering from DCM (30). The interaction of striatins with Slmap is even viewed as a critical regulator of the cardiac excitation-contraction machinery (42, 43).

Moreover, the STRIPAK-associated phosphatase Pp2a has been linked to ID protein dephosphorylation events for more than 20 years (44, 45). Despite years of further research, the knowledge of kinases involved in the regulation of the ID is still incomplete. Here, we now show for the first time that various STRIPAK proteins are abundantly expressed in the heart and that core STRIPAK members striatin, Slmap, Strip1, and Strip2/Myoscape as well as the mammalian sterile20-like kinase Mst4 interact within cardiomyocytes. In other cell types, STRIPAK-associated Mst4 is thought to antagonize PP2A phosphatase activity and is itself negatively regulated by PP2A-dependent dephosphorylation at T178 (*via* its activating site by autophosphorylation, Mst3-dependent, and PKA-dependent phosphorylation). Autophosphorylation not only results in increased activity but also results in homodimer formation crucial for kinase function (6, 7, 15). Another negative regulator seems to be the STRIPAK adaptor protein Strip1, which itself is competitively inhibited by Strip2/Myoscape in various cell types. Association of Mst4 to substrates, adaptors, and binding partners results in different subcellular localizations of the kinase (15). This further illustrates that Mst4 functions and the effects of STRIPAK must always be seen as an interaction of kinases and phosphatases and possibly other associated proteins.

In subsequent experiments, we could show that Mst4 is differentially regulated under various cardiac stress conditions, potentially indicating important physiological functions to maintain cardiac homeostasis. We observed marked Mst4 upregulation in human cardiomyopathy and various rodent models of cardiomyopathy *in vivo* as well as after increasing cellular densities (and thereby cell–cell contacts) in cardiomyocyte culture *in vitro*. This could denote a possible pathophysiological role in these settings or, since we have observed various rather protective effects of Mst4 overexpression *in vitro*, an adaptive response. Since ID and cell–cell contacts also tend to mature during development (46), this could also play a role in increasing Mst4 protein abundance.

In cardiomyocytes, Mst4 locates to perinuclear regions (possibly the golgi apparatus as it has been shown in other cell types (47, 48)) and stress fibers. In human myocardium, it predominantly appears at IDs colocalizing with ß-catenin. The localization of Mst4 at the ID seems particularly plausible, since a large number of the interaction partners we found, such as striatins, Slmap, ß-catenin, alpha-actinin2, desmin, desmoglein, and plakophilin, but also a number of the potential kinase targets, such as connexin 43, Plm, desmin, Ehd2, junction plakoglobin, Zo-1, and the sodium/hydrogen exchanger 1 localize to the ID (26, 28, 40, 49, 50). The role of phosphorylation at the ID in various cardiac diseases has also been investigated, although the exact associated kinase has not always been identified (31, 32, 51). By comparing different patient samples from patients with cardiomyopathy and healthy subjects and performed phosphoproteomic analysis, Reitz and colleagues were able to identify alpha-catenin as a phosphorylation target in this disease and a crucial role for phosphorylated catenin in ID integrity and proper cardiac function (32). In a mouse model of pressure overload, Chang and colleagues identified phosphorylated dynamin-related protein 1 in phosphoproteomic analyses as a critical regulator of hypertrophy at the ID (51). Taken together, the ID in its complex three-dimensional structure and integrity appears to be subject to pronounced adaptations in heart failure and cardiomyopathy, including both its architecture and associated signaling pathways (34).

We identified Plm and the beta subunit of LTCC, both located to the cardiac sarcolemma, as potential Mst4 kinase substrates. The beta subunit of LTCC is already known to be regulated by PKA-dependent phosphorylation at T164 and S591 (52). Mst4 thereby phosphorylates S510 of rat voltage-dependent LTCC subunit beta-2. Whether this phosphorylation affects LTCC function and/or surface retention on the t-tubule membrane has to be addressed in future experiments. The serine at position 83 (S83) of Plm, which was hyperphosphorylated in our phosphoproteomics study, corresponds to S63 in most publications because the first 20 amino acids act as a signal peptide. Plm is a cardiac transmembrane protein associated with ionic currents and is a substrate for several kinases. Known regulators are PKA and PKC (PKC mainly α and ε isoforms), whereas STRIPAK-associated PP2A is described as a major player in the dephosphorylation of S63 (50, 53). The expression and phosphorylation pattern of Plm is altered in various cardiac diseases (53). Interestingly, phosphorylation of connexin43 at serine 262 (S262) has been described for PKC. MAP kinase Erk1/2 and p38-dependent phosphorylation of C × 43 was associated with cardiomyocyte cytoprotection and enhanced DNA synthesis. Dephosphorylation of C x 43 occurs in cardiac ischemia and is believed to contribute to the detrimental effects, mediated also *via* PP2A (44, 45).

While Mst1 has been linked to promote muscular atrophy in skeletal muscle, we could show that Mst4 promotes physiological hypertrophy in cardiomyocyte culture. This was underlined by increased phosphorylation of the PKB (Akt) (54) and the absence of the induction of markers of pathological hypertrophy like the fetal gene program or increased CnA activity. Moreover, increased Akt phosphorylation was only seen after 24 h of overexpression and absent after 48 h of adenoviral treatment. This would also explain the fact that Akt was not identified as a possible target in our phosphoproteomics approach, comparing 48 h and 72 h of Mst4 overexpression. Given the fact that Mst1/2 are typically counter regulated by Mst3/4, suppression of Mst1 function by Mst4 could contribute to these observed prohypertrophic effects. Overall, our results identify Mst4 as prohypertrophic and procontractile kinase that protects from cardiac apoptosis. This is in line with work from Xiong *et al.* showing that Mst4 activity promotes protection from hypoxia-induced apoptosis in pituitary tumors, an effect that was abolished by hesperadin (55, 56). Specifically, our results also indicate that the prosurvival effect of Mst4 is dependent on kinase function rather than on gene regulation.

The ID itself is also a crucial cardiac structure in mechanoperception and signal transduction, both in terms of sensing mechanical forces in myocardial tissue and in transmitting and fine-tuning the cardiomyocyte response to mechanical forces (49, 57). On the other hand, it is also likely that Mst4-dependent phosphorylation affects the function of the ID in terms of mechanotransduction and cardiac contractility. A fine-tuned interplay between STRIPAK kinase–dependent phosphorylation and STRIPAK phosphatase–dependent dephosphorylation is likely and needs to be further elucidated in future experiments. The observed interaction with Serca2a and also potential phosphorylation of ryanodine receptors might interplay and offer short-term and long-term fine-tuning options in various physiological and pathophysiological conditions.

In summary, we here show for the first time that STRIPAK is present in the heart with differentially regulated core protein members under various pathologic conditions. Cardiac Mst4 kinase is highly enriched in heart and skeletal muscle and is significantly induced in human cardiomyopathy. Functionally, Mst4 acts as a prohypertrophic, procontractile, and prosurvival kinase likely by phosphorylating several cardiac targets along the ID, sarcomeric Z-disc, and ion channels within the cardiac dyad. Further *in vivo* and *in vitro* experiments will clarify if and under which specific conditions these phosphorylation events occur and what the downstream effects are.

### Limitations of the study

Although a great deal of effort and care has been taken, the results of the phosphoproteomics studies must still be regarded as preliminary. Useful commercial and phosphorylation site-specific antibodies do not yet exist on a large scale. The changes and signaling pathways triggered by any phosphorylation must also be investigated further in order to examine precise Mst4-dependent mechanisms and to better understand the complex interplay of STRIPAK-dependent kinases and phosphatases. Furthermore, it should be noted that the healthy myocardium used for this study derives from patients after heart transplant undergoing routine biopsies in accordance to ISHLT standards. These patients are required to be treated with immunosuppressants that could potentially affect Mst4 expression, but acquiring samples from other healthy hearts would be highly unethical.

## Experimental procedures

### Cloning of Mst4 and generation of a recombinant adenovirus

AdMST4 was generated using the ViraPower Adenoviral Expression System (Invitrogen) according to manufacturer’s instructions and as previously described (5). In brief, Mst4 was cloned from rat heart complementary DNA (cDNA) using primers (5′-GCTGGCACCATGGCCCACTCACCGG-3′ and 3′-GCTGGGTCGCCGTTAAGGGGATTCATCCGCG-5′) in pDonR221 gateway cloning vector. This cDNA in pDonR221 vector was transferred into the pAd/CMV/V5-DEST destination vector. These constructs then were digested with PacI restriction enzyme and transfected into HEK293A cells to produce respective protein-expressing adenoviruses. Titration for the viruses was performed by staining virus-infected HEK293A cells with fluorescent anti-hexon antibody. A ß-galactosidase-V5–encoding adenovirus (Ad-LacZ; Thermo Fisher Scientific) served as a control.

### Isolation and culture of NRVCMs

NRVCMs were prepared as described previously (5). In brief, for isolation of NRVCMs, left ventricles from 1- to 2-day old Wistar rats (Charles River) were harvested and chopped in ADS buffer (116 mM NaCl, 19.7 mM Hepes, 9.4 mM NaH_2_PO_4_∙H_2_O, 5.55 mM glucose, 5.36 mM KCl, 0.83 mM MgSO_4_). For the release of individual cardiomyocytes from chopped tissue mass, 1 ml per heart of digestion buffer (ADS buffer containing 0.5 mg/ml collagenase type II and 0.6 mg/ml pancreatin) was added and incubated for 20 min. Cell suspension was then passed through a cell strainer, followed by the addition of 1 ml newborn calf serum per 5 ml cell suspension to stop enzymatic digestion. Digestion was repeated 2 to 3 times with remaining tissue suspension to increase yield. Cardiomyocytes were separated from fibroblasts using a Percoll gradient (GE Healthcare) centrifugation step and cultured in Dulbecco's modified Eagle's medium (DMEM) (gibco) containing 10% fetal calf serum, 100 U/ml penicillin G, 100 μg/ml streptomycin, and 2 mM L-glutamine (PAA Laboratories).

### Overexpression of Mst4 in NRVCM

For MST4 overexpression, adenovirus infection with 25 ifu/cell in DMEM supplemented with penicillin/streptomycin and L-glutamine but lacking fetal calf serum was performed 24 h postisolation. Medium was changed 48 h postinfection, and cells were harvested 72 h postinfection for further analysis.

### Knockdown of Mst4 in NRVCM

Silencer select predesigned siRNA (Thermo Fisher Scientific) was used for Mst4-knockdown (siMST4 ID s165383, siMST4 ID s165382, siRNA negative control No. 1, GAPDH positive control siRNA). The siRNA and lipofectamine RNAiMAX (Thermo Fisher Scientific) was prepared in DMEM with L-glutamine only according to manufacturer’s instructions. Twenty-four hours postisolation, cells were transfected with 5 mM of siRNA, medium was changed 48 h posttransfection, and cells were harvested 4 days posttransfection for further analysis.

### RNA isolation and quantitative real time PCR

Total RNA was isolated from NRVCMs or mouse heart samples using Quick-RNA Microprep Kit (Zymo Research) following the manufacturer’s instructions. One microgram of DNA-free total RNA was transcribed into cDNA using the LunaScript RT SuperMix Kit (New England BioLabs). For quantitative real time PCR (qRT-PCR), the SensiFAST SYBR Green Mastermix reagent (Meridian Bioscience) was used in a real-time PCR system (CFX96; Bio-Rad). Cycling conditions used for all the SYBR-qRT-PCRs were 2 min at 95 °C, followed by 40 cycles of 5 s at 95 °C and 30 s at 60 °C, a common step for annealing and elongation at which step data were collected. Rpl32 or RPLP0 ribosomal RNA genes were used as an internal standard for normalization. Multiplex qRT-PCR for parallel measurement of NPPA-, NPPB-, RCAN1.4-, and RPL32-cDNA was performed using Bio-Rad iQ Multiplex Powermix. Cycling conditions were 2 min at 95 °C, followed by 40 cycles of 15 s at 95 °C and 45 s at 60 °C. All experiments with NRVCMs were performed in triplicate and repeated three times.

### Primers used for qRT-PCR

See Table “Experimental procedures 1 (Primers used for qRT-PCR)” (Table 1).

**Table 1:** **Primers used for qRT-PCR:**

Gene	Species		Sequence
MST4	mouse, human, rat	forward	CCCTCAGAGAGTCATGGACC
		reverse	CGGGGTCAACTTGTCATCTT
RPL32 SYBR	Mouse, rat	forward	GGTGGCTGCCATCTGTTTTACG
		reverse	CCGCACCCTGTTGTCAATGC
VEGFA	rat	forward	CAGGCTGCACCCACGACAGA
		reverse	GACGGCAATAGCTGCGCTGG
RPLP0	rat	forward	ATCTCCCCCTTCTCCTTCGGGC
		reverse	CAGGGCCTGCTCTGTGATGTCC
NPPA Multiplex	Mouse, rat	forward	GGAGCAAATCCTGTGTACAGTG
		reverse	ACCTCATCTTCTACCGGCAT
		probe	FAM-TGATGGATTTCAAGAACCTGCTAGACCA-BHQ1
NPPB Multiplex	rat	forward	AGAAGATAGACCGGATCGGC
		reverse	AGCCAGGAGGTCTTCCTAAA
		probe	HEX-TCAGCCCGTCACAGCCCAAGCGA-BHQ1
RCAN 1.4 Multiplex	Mouse, rat	forward	TAGCTCCCTGATTGCTTGTG
		reverse	GGATTCAAATTTGGCCCTGG
		probe rat	CY5.5-ACGATGATGTCTTCAGCGAAAGTGAGAC-ECL
		probe mouse	CY5-ACGATGATGTCTTCAGCGAAAGTGAGAC-BHQ2
RPL32 Multiplex	Mouse, rat	forward	CTGCTGATGTGCAACAAATCT
		reverse	GCTGTGCTGCTCTTTCTACAAT
		probe	RED-ACTGTGCTGAGATTGCTCACAATGTGT-BHQ2
Striatin	Mouse, rat	forward	CTCAGGCAGTATCTCCAGGAGG
		reverse	TTCCATCAGTGTCCTCGTCC
Striatin 3	Mouse, rat	forward	CCCTATGATACATATGAGTCAAACG
		reverse	ATGGCAGCTTTTCTTGTGGG
SMLAP	Mouse, rat	forward	GCTCTGCAAGTACGGTTAGAAC
		reverse	AATAAAAGTGCAGTCCCCGC
STRIP1	Mouse, rat	forward	CTTCCGGATCCATGTGTCAGA
		reverse	AGCTGCACTCTCCAAAGGTA
CCM3	Mouse, rat	forward	GAACCGCAGGGCACTTGAA
		reverse	CAGGCCACAGTTTTGAAGGT
STRIP1	Mouse, rat	forward	CTTCCGGATCCATGTGTCAGA
		reverse	AGCTGCACTCTCCAAAGGTA
MST3	Mouse, rat	forward	CAGAGATATTAAAGCGGCCAATG
		reverse	TTTGGCCAGTTCTATTGCGG

### Protein preparation and immunoblotting

NRVCMs were washed with ice cold PBS and lysed by three freeze-thaw cycles in radioimmunoprecipitation buffer (1% NP-40, 1% sodium deoxycholate, 0.1% SDS, 150 mM NaCl, 10 mM sodium phosphate buffer (pH 7.2), 1 mM DTT, phosphatase inhibitor II, phosphatase inhibitor III, protease inhibitor mixture (Roche Applied Science)). Cell debris was removed by centrifugation, and protein concentration was determined photometrically by detergent compatible assay method (Bio-Rad). Protein from mouse and human hearts was harvested by homogenizing heart fragments along with Precellys ceramic beads (Peqlab) in radioimmunoprecipitation buffer. Protein samples were resolved by 10% SDS-PAGE, transferred to a nitrocellulose membrane, and immunoblotted with the target-specific primary antibodies. The overnight application of primary antibodies was followed by incubation with a suitable horseradish peroxidase-coupled secondary antibody (1:10,000; Dianova) or fluorescent antibody Alexa Fluor 546 (1:2000; Thermo Fisher Scientific). Finally, visualization was achieved using a chemiluminescence kit (GE Healthcare) and was detected on an imaging system (FluorChem Q; Biozym). Quantitative densitometry was performed using ImageJ version 1.53a software (National Institutes of Health, imagej.net).

### Primary antibodies

See Table “Experimental procedures 2 (Primary antibodies)” (Table 2).

**Table 2:** **Primaryantibodies**

Target protein	Species, clonality	Manufacturer	Reference	Dilution
Akt	Rabbit, poly	Cell Signalling	#9272	WB 1:1000
Akt, Phospho-S473 (S473)	Rabbit, poly	Cell Signalling	#9271	WB 1:1000
Caspase3	Rabbit, poly	Cell Signaling	#9662S	WB 1:1,000
Caspase3, cleaved	Rabbit, mono	Cell Signaling	#9664	IF 1:400
Caspase7	Rabbit, poly	Cell Signaling	#9492S	WB 1:1,000
b-Catenin	Rabbit, poly	Invitrogen	71-2700	WB 1:1000 IF 1:100
GAPDH	Mouse, poly	Sigma	G8795	WB 1:10,000 Co-IP 1:100
MST4	Rabbit, poly	Cell Signaling	#3822	WB 1:1,000 IF 1:100
MST4	Rabbit, mono	Abcam	ab52491	WB 1:1,000 Co-IP 1:100 IF 1:250
MST4	Mouse, mono	Abnova	H00051765-M02	IF 1:25
MST4, Phospho-T178	Rabbit, mono	Abcam	ab76579	WB 1:1,000
MST3	Rabbit, poly	antibodies-online	ABIN7301338	WB 1:1000
PARP	Rabbit, poly	Cell Signaling	#9542	WB 1:1,000
VEGFA	Rabbit, poly	Abcam	ab46154	WB 1:1,000
α-Actinin 2	Mouse, mono	Sigma	A7811	WB 1:1000 IF 1:200
α-Tubulin	Mouse, mono	Sigma	T5168	WB 1:8,000
Cav 1.2	Mouse, mono	Origene	TA309306	IF 1:100
SERCA2A	Mouse, mono	Santa Cruz	sc-376235	WB 1:1000
Striatin	Rabbit, poly	Origin	TA301717	WB 1:1000
STRIP1	Mouse, mono	Novus Biologicals	NBP2-45715	WB 1:1000
STRIP2	Rabbit, poly	proteintech	25163-AP	WB 1:1,000
SLMAP	Rabbit, poly	Novus Biologicals	NBP1-81398	WB 1:1,000
STRN3	Mouse, mono	Novus iologicals	NB110	1:1000
CCM3	Rabbit, poly	Abcam	ab180706	WB 1:100

### Coimmunoprecipitation

Ten million isolated NRVCMs were plated on 10-cm dishes, cultivated, harvested with erythrocyte lysing buffer (50 mM Hepes, 250 mM NaCl, 5 mM EDTA, 1% NP-40), lysed, and cell debris was removed by centrifugation. After protein concentration measurement by detergent compatible assay (Bio-Rad), fractions with 500 μg of protein in 500 μl erythrocyte lysing buffer each were prepared. Fractions were either incubated with antibodies targeting Mst4 or Gapdh/Ig (negative control) or no antibody (negative control) over night at 4 °C. Thirty microliters of magnetic Dynabeads (Thermo Fisher Scientific) were added and incubated for 4 h at 4 °C. Nonbound protein was removed in five washing cycles. Samples were placed on a magnetic rack, supernatant was removed, erythrocyte lysing buffer was added, and incubated for 5 min at 4 °C. They were eluded in 50 μl Laemmli buffer and incubated for 5 min at 95 °C. Dynabeads were removed using the magnetic rack, and supernatant was transferred into a new cup. Ten microliters of each sample as well as an untreated or control treated fraction of the input (positive control) were used for immunoblotting using either then desired detection antibodies.

### Immunofluorescence microscopy

Immunofluorescence microscopy was used for intracellular localization of Mst4, cell surface area measurements, and cleaved caspase 3 staining. Therefore, either 180,000 NRVCMs were seeded on collagen-coated coverslips in 12-well plates or heart tissue was embedded in Tissue-Tek (Sakura), frozen on dry ice and sliced to a thickness of ca. 6 μm. Cells or slices were washed with ice cold PBS and fixed with 4% (v/v) paraformaldehyde (Sigma-Aldrich) for 10 min, permeabilized, and blocked with 0.1% (v/v) Triton X-100 (Sigma-Aldrich) and 2.5% (w/v) bovine serum albumin (BSA) for 1 h at room temperature. Afterward plates were incubated with primary antibodies over night at 4 °C. Following dilutions in 2.5% (w/v) BSA in PBS were used: anti-Mst4 (1:100, polyclonal, rabbit, Cell Signaling #3822), anti-ACTN2 (1:200, monoclonal, mouse, Sigma A7811), and anti-cleaved-Casp3 (1:400, monoclonal, rabbit, Cell Signaling #9664). Cover slips were washed with PBS and incubated for 1 h in diluted secondary antibody and DAPI (1:5000, Roche 10236276001) at room temperature. Antibodies coupled to Alexa Fluor (AF) dyes served as secondary antibodies: anti-mouse (1:500, AF546, polyclonal, donkey, Thermo Fisher Scientific A10036), anti-mouse (1:500, AF488, polyclonal, chicken, Thermo Fisher Scientific A21200), and anti-rabbit (1:400, AF546, polyclonal, donkey, Thermo Fisher Scientific A10040). FluorPreserve Reagent (Calbiochem) was used to fix coverslips on microscope slides. Immunofluorescence micrographs for intracellular localization were captured by confocal microscope (Carl Zeiss LSM 800).

### Immunofluorescence in paraffin-embedded human heart sections

Human heart paraffin-embedded sections were collected from stable patients post heart transplants with normal heart functions and no immunohistological signs for transplant rejection. Samples were provided by the Tissue Bank of the National Centre for Tumor Diseases in accordance with the regulations of the tissue bank and approval of the ethics committee Heidelberg University.

After rehydration with xylene and decreasing concentrations of ethanol, sections were washed with PBS. Antigen retrieval was performed with sodium citrate buffer (pH 6), including heating up to 100 °C and placing the samples on dry ice. Sections then were washed with PBS and were incubated for 10 min in 0.1% Triton X-100 for permeabilization. Blocking was performed by using blocking buffer: 2.5% (w/v) BSA in PBS and 1 h incubation in a humidity chamber.

Primary antibodies were placed overnight in the desired concentration in blocking buffer. Secondary antibodies were used in blocking buffer for 1 h at room temperature. Between each step, sections were rinsed with PBS 3 × 5 min. Then, samples were stained with DAPI and mounted with mounting medium prior to microscopy analysis.

### Cell size (surface area) measurements

5 × 5 × 3 (xyz) images of each coverslip were taken at 200x magnification (Nikon; CFI Plan Apochromat), *z*-stack mode and with a *z*-pitch of 3.0 μm by a Keyence BZ-9000 fluorescence microscope. Using BZ-II Analyzer, pictures were merged and a full-focus calculation was performed. HybridCellCount software was used for cell surface measurement, and fluorescence intensity was determined in single-extraction mode. After setting thresholds for a reference picture, MacroCellCount was performed of each picture and the counted cells were filtered (exclusion of cells with more than one nucleus, smaller 150 μm^2^, or greater 3000 μm^2^).

### Cleaved caspase 3 measurement

NRVCMs were isolated and stained with antibody-targeting cleaved caspase 3 and DAPI as described above. Ten micrographs of each coverslip were taken at 200x magnification (Nikon; CFI Plan Apochromat) by a Keyence BZ-9000 fluorescence microscope. Using BZ-II Analyzer, dots representing cleaved caspase 3 and nuclei were counted and put into ratio.

### Isolation and culture of ARVCMs

Six-week-old Wistar rats were narcotized using isoflurane. The chest was opened, 20 IE heparin was injected into inferior vena cava, thymus was removed, and aorta was exposed. The heart was grabbed with forceps, transverse aorta, as well as lung vessels were cut and the heart was transferred into ice cold PBS.

After inserting and securing a buttoned cannula into the aorta above the aortic valve, coronary arteries were carefully flushed with isolation buffer (120.4 mM NaCl, 14.7 mM KCl, 0.6 mM KH_2_PO_4_, 0.6 mM NaH_2_HPO_4_∙H_2_O, 1.2 mM MgSO_4_∙7H_2_O, 10 mM Hepes, 4.6 mM NaHCO_3_, 30 mM taurine, 10 mM 2,3-butanedione monxime, 5.5 mM Glucose). The heart was inserted into a Langendorff apparatus (Bochem Instrumente) and perfused for 15 to 20 min at a rate of 8 ml/min with digestion solution (50 ml isolation buffer containing 90 mg collagenase II (Worthington, 260–290 U/mg) and 40 μM CaCl_2_) that was recycled within the apparatus.

Aorta and atria were removed and using scissors the tissue was dissected. Five milliliters stop buffer (isolation buffer containing 1% (w/v) BSA and 12.5 μM CaCl_2_) were added to end digestion and cells were suspended by pipetting up and down for 2 to 3 min until no tissue was visible. Cell suspension was transferred into 50-ml tube through a 200-μm cell filter.

For calcium toleration, cells were resuspended in ca. 25 ml stop buffer with increasing concentrations of calcium (100 μM, 400 μM, 900 μM) for 10 min each. Cell concentration was counted, adjusted to 100 to 200 cells per microliter, and 40,000 to 50,000 cells in 100 to 300 μl were plated on laminin-coated square coverslips in 6-well-plates for IonOptix analysis, directly on 6-well plates for protein preparations or round coverslips for immunofluorescence. After 1 h cells were either fixed or 2 ml of ARVCM medium (medium 199 with Earles salts, NaHCO₃ containing 10 mM creatine, 20 mM Taurine, 1% (w/v) BSA, 100 U/ml penicillin G, 100 μg/ml streptomycin, and 2 mM L-glutamine) with virus (500 ifu/cell) and/or inhibitor Hesperedin (100 mM) were added.

### Analysis of contraction and calcium cycling using IonOptix system

Isolated ARVCMs were plated on square coverslips, 500 multiplicity of infection virus and/or 100 nM inhibitor were added and cells were incubated for 24 h (20% O_2_, 5% CO_2_, 37 °C, 85% humidity). Medium was taken off, fresh ARVCM medium containing 2 μM Fura-2AM was added, cells were incubated for 20 min, medium was changed to Fura-2AM–free medium, and cells were incubated for another 20 min.

Using the IonOptix system, cardiomyocytes were stimulated (2 Hz, 10 V, biphasic pulse) and several cells per coverslip were recorded. These recordings were analyzed using IonOptix software (ionoptix.com) for calcium cycling, cellular as well as sarcomeric contraction and several parameters per cell were exported for statistical analysis.

### MLP-, CS1-KO, and CnA-TG mice

All genetically modified mice were generated from C57BL/6N inbred mice. MLP-KO mice were generated by Arber *et al.* 1997, CS1-KO mice by Frey *et al.* 2004, and CnA-TG mice by Molkentin *et al.* 1998. For this publication, already existing samples were used.

### Transverse aortic constriction

Transverse aortic constriction was performed in 8-week-old C57BL/6N mice (Charles-River Laboratories). They were anesthetized with combination of ketamine (120 mg/kg i.p.) and xylazine (15 mg/kg i.p.). The mice were then orally intubated with a 20-gauge tube and ventilated (Harvard Apparatus) at 120 breaths per min (0.2 ml tidal volume). The aortic constriction was performed *via* a lateral thoracotomy through the second intercostal space. A suture (Prolene 6–0) was placed around the transverse aorta between the brachiocephalic and left carotid artery. The suture was ligated against a 27-gauge needle. The needle was later removed leaving a discrete stenosis. The chest was sutured, and the pneumothorax was evacuated. Sham-operated animals underwent the same procedure except for ligation. Cardiac function was examined by echocardiography, and the animals were killed 2 weeks postimplantation to extract the heart for downstream applications. All the animal experiments were approved and performed as per the guidelines of local ethical committee (Ministerium für Energiewende, Landwirtschaft, Umwelt, and Ländliche Räume Schleswig-Holstein).

### Human heart samples

Left ventricular myocardial tissue was taken from explanted hearts of 19 patients (10x DCM, 9x ischemic cardiomyopathy) with end-stage heart failure (NYHA IV) undergoing heart transplantation. Control samples were taken from transplanted hearts in routine examinations (6x nonfailing control myocardium). All procedures involving humans were performed in compliance with the ethical committee of the medical faculty of the Georg-August University. The explanted hearts were acquired directly in the operating room during surgical procedures, frozen in liquid nitrogen, and stored at −80 °C immediately after excision.

### Phosphoproteomics analysis

Using the SysQuant phosphoproteomic workflow (Proteome Sciences) six experimental groups with three replicates each of NRVCMs were examined.

See Table “Experimental procedures 3 (Phosphoproteomics analysis)” (Table 3).

**Table 3:** **Phosphoproteomics analysis**.

	LacZ 48 h	MST4 48h	Untreated 72 h	LacZ 72 h	MST4 72 h	MST4 + Hes 72 h
AdLacZ (moi)	50	0	0	50	0	0
AdMST4 (moi)	0	50	0	0	50	50
Time (h)	48	48	72	72	72	72
Hesperadin (nM)	0	0	0	0	0	100

Using the SysQuant phosphoproteomic workflow (Proteome Sciences, London) six experimental groups with three replicates each of NRVCMs were examined.

NRVCMs were isolated, 10 million plated in 10-cm dishes each, and treated as described above. Forty-eight or seventy-two hours after infection, cells were washed three times with ice cold PBS, 600 μl of SysQuant buffer (8 M urea, 50 mM Tris–HCl (pH 7.6), 75 mM NaCl in water, pH adjusted to 8.2. Per 10 ml buffer 1 tablet complete Mini protease inhibitor (Roche) and one tablet PhosSTOP (Sigma-Aldrich)) were added, cells were briefly frozen at −80 °C, and transferred into a 1.5ml-cup. Samples were centrifuged for 20 min (14,000*g*, 4 °C), supernatants were transferred into new cups and sent to Proteome Sciences. Quality control and concentration measurement was performed and samples at equal concentrations were created. They were reduced using DTT, alkylated using iodoacetamide, digested into peptides using trypsin, and tandem mass–tagged for MS. Samples were pooled in two TMT11plex, and phosphoproteomics analysis was performed according to the company’s standards. Detailed protocols can be requested from the authors.

After using exploratory analysis to build up the most appropriate model for finding differentially regulated features, linear modeling using R package LIMMA (Proteome Sciences) (bioinf.wehi.edu.au/limma/) was applied to find out regulated peptides and proteins. The significance criterion of α was set according to guidance provided by volcano plots to identify significantly regulated features. Multiple testing corrections were performed using the Benjamini–Hochberg procedure. In addition to volcano plots, principal component plots were generated to display the results of feature selection. For reproducibility of features regulation measurement a log fold change threshold was set to exceed twice the median total variance. Log2 fold changes were calculated for all the contrasts for all features. Statistics was calculated on peptide, phosphopeptide, and protein levels. Results were visualized in heatmaps.

Functional analysis was performed to identify GO terms, pathways, and kinase substrates that are significantly altered between the experimental groups, where the thresholds set for feature selection were used to select regulated peptides (*p* < 0.05; |fold change|>log2(1)). A Significance of Enrichment analysis, based on the Fisher Exact Test, was performed by means of a tool developed by the company (functional analysis toolv1.2.0). Enrichment of functional terms: GO biological processes; GO molecular functions, pathways, and kinase substrates were performed within functional analysis tool. A two-sided *p* value was generated by the Fisher exact test and the Benjamini–Hochberg method was used for multiple test correction. A minimum of two matched identifiers (*e.g.*, gene names) was required and terms with an adjusted significance value <0.3 were considered significant. All functional results were visualized using volcano plots (enrichment *versus* adjusted *p* value).

### Co-IP with LC-MS/MS analysis

Each eluant (n = 2) was separately run on the SDS-PAGE for 1 to 1.5 cm. Three 0.5 cm gel slices were collected from each lane and were washed once with 60 μl of 1:1 (v/v) 50 mM triethylammonium bicarbonate buffer (TEAB; Sigma-Aldrich) and acetonitrile (ACN), pH 8.5 for 10 min, shrunk three times for 10 min each in 60 μl ACN, and washed in 60 μl 50 mM TEAB, pH 8.5. Following a reduction of proteins with 10 mM DTT (Sigma-Aldrich) in 100 mM TEAB at 57 °C for 30 min and dehydration of gel pieces, proteins were alkylated with 10 mM IAA (Sigma-Aldrich) in 100 mM TEAB at 25 °C for 20 min in the dark. Prior to protein digestion, gel pieces were washed with 60 μl 100 mM TEAB and shrunk twice for 10 min in 60 μl ACN. A total of 30 μl of 8 ng/μl in 50 mM TEAB trypsin solution (sequencing grade, Thermo Fisher Scientific) was added to the dry gel pieces and incubated 4 h at 37 °C. The reaction was quenched by addition of 20 μl of 0.1% TFA (Biosolve). The resulting peptides were extracted once for 30 min with 30 μl 1:1 (v/v) 0.1% TFA and ACN, followed by gel dehydration with 20 μl ACN for 20 min, and washed with 30 μl of 100 mM TEAB for another 20 min. Finally, gel was shrunk twice with 20 μl of ACN for 20 min. The supernatant from each extraction step was collected and dried in a vacuum centrifuge. Three gel fractions were dissolved in 15 μl 0.1% TFA each and combined in one well before the LC-MS analysis.

### LC-tandem mass spectrometry analysis

Nanoflow LC-tandem mass spectrometry analysis was performed with a Vanquish Neo ultrahigh performance liquid chromatography system coupled to an Orbitrap QE HF (Thermo Fisher Scientific). An in-house packed analytical column (75 μm × 200 mm, 1.9 μm ReprosilPur-AQ 120 C18 material [Dr Maisch]) was used. Mobile phase solutions were prepared as follows, solvent A: 0.1% formic acid, solvent B: 0.1% formic acid, 80.0% ACN. Peptides were separated in a 60 min linear gradient started from 3% B and increased to 26% B over 50 min and to 43% B over 10 min, followed by washout with 99% B. The mass spectrometer was operated in data-dependent acquisition mode, automatically switching between MS and MS2. MS spectra (*m/z* 400–1600) were acquired in the Orbitrap at 60,000 (*m/z* 400) resolutions and MS2 spectra were generated for up to 15 precursors with normalized collision energy of 27 and isolation width of 1.4 *m/z*. The tandem mass spectrometry spectra were searched against the SwissProt *Rattus norvegicus* (UP000002494, November 2019) protein database and a customized contaminant database (part of MaxQuant, MPI Martinsried) using Proteome Discoverer 2.5 (https://www.thermofisher.com/order/catalog/product/OPTON-31099) with Sequest HT (Thermo Fisher Scientific). A fragment ion mass tolerance was set to 0.02 Da and a parent ion mass tolerance to 5 ppm. Trypsin was specified as enzyme. Carbamidomethylation was set as fixed modification of cysteine, and oxidation (methionine) and deamidation (asparagine, glutamine) as variable modifications of peptides. Acetylation, methionine loss, and combination of acetylation and methionine loss were set as variable modifications of protein terminus. Peptide quantification was done using precursor ion quantifier node with Top N Average (n = 3) method set for protein abundance calculation. Only proteins identified with at least two peptides and assigned as master proteins were used for the analysis. Protein intensities were normalized to the samples’ median. Protein quantification in at least one sample group (n = 2) was used as a further filtering criterion. Missing data was imputed (n = 2) using random draws from a manually defined left-shifted Gaussian distribution. Next, the ratio between the corresponding MST4 and LacZ samples were calculated (see Supplementary table). The ggplot R package was employed for data visualization.

### Statistical analyses

All results are shown as the mean ± SD unless stated otherwise. Real time PCR data analyses were carried out using the ΔΔ^ct^ method. Analyses for normal distribution were performed by Shapiro-Wilk test. Statistical analyses of the data were carried out using one-way ANOVA, followed by Student-Newman-Keuls post hoc tests. If appropriate, Student’s *t* test (two tailed, unpaired) was employed. *p* values <0.05 were considered statistically significant.

## Data availability

The authors are committed to transparency and reproducibility in research. Access to the data allows for verification, replication, and further analyses by other researchers. For inquiries or additional information, please contact the corresponding author.

## Supporting information

This article contains supporting information.

## Conflict of interest

The authors declare that they have no conflicts of interest with the contents of this article.
