# Supplementary material for: Mst4, a novel cardiac STRIPAK complex–associated kinase, regulates cardiomyocyte growth and survival and is upregulated in human cardiomyopathy
Source: J Biol Chem. 2024 Apr 3;300(5):107255. doi: 10.1016/j.jbc.2024.107255 (PMC11087964; doi:10.1016/j.jbc.2024.107255)
Supplement: Supporting Information [file mmc1.docx]

# Supporting Information:

## Supporting Figure 1:

(A) Immunoblotting experiments comparing Mst4 protein abundance in mice with cardiac restricted transgenic expression of the pro-hypertrophic phosphatase Calcineurin (CnA-TG; n=5) compared to wildtype littermates (WT; n=5) (t-test: p=0.0001). (B) Immunoblotting experiments comparing Mst4 protein abundance in mice after transverse aortic constriction (TAC; n=5) compared to appropriate sham operated animals (n=5) (t-test: p<0.0001). (***p<0.001; ****p<0.0001).

## Supporting Figure 2:

Increased MST4 (t-test: p=0.015) and also NPPA, NPPB and RCAN 1.4 mRNA abundance (t-tests: p<0.0001) in NRVCMs subjected to bidirectional cyclic stretch (Flexcell systems) for 48h and compared to non-stretched control conditions. (n=4 experiments with 3-6 biological replicates each); * p<0.05; ****p<0.0001)

## Supporting Figure 3:

(A) Assessment of global calcium transients by FURA2-AM loading in ARVCM after Mst4 overexpression and co-incubation with hesperadin compared to LacZ and vehicle controls. Diastolic (A) systolic (B) and transients (C) are displayed. Also, the time to peak concentrations and the influx and efflux velocities of calcium transients were analyzed (D-H), showing significant impairment of calcium cycling after hesperadin treatment. (* p<0.05; ** p<0.01).

## Supporting Figure 4:

Table of selected enriched phosphoproteins and phosphorylation sites after 48h and 72h of Mst4 overexpression compared to LacZ and parallel hesperadin treatment (72h AdMST4+hesperadin).

## Supporting Figure 5:

Tables showing enriched gene ontology (GO) terms after 48h Mst4 overexpression (upper table) and 72h Mst4 overexpression (bottom) compared to LacZ control.

## Supporting Figure 6:

Phosphopeptide level GO molecular function (GOMF) enrichment for 48h of Mst4 overexpression. Volcano plot showing log2 enrichment against significance of enrichment, highlighting the MFs found to be significantly over- (right side of zero) or under- (left of zero) represented by the differentially-expressed peptides in the data set. Filled red circles highlight significant hits (FDR<0.3) with the size related to the corresponding rank value.

## Supporting Figure 7:

Phosphopeptide level GO biological process (GOBP) enrichment for 48h of Mst4 overexpression. Volcano plot showing log2 enrichment against significance of enrichment, highlighting GO terms for biological processes found to be significantly over- (right side of zero) or under- (left of zero) represented by the differentially-expressed peptides in the data set. Filled red circles highlight significant hits (FDR<0.3) with the size related to the corresponding rank value. Protein phosphorylation was found to be highly significantly upregulated.

## Supporting Figure 8:

Phosphopeptide level GO molecular function (GOMF) enrichment for 72h of Mst4 overexpression. Volcano plot showing log2 enrichment against significance of enrichment, highlighting GO terms for molecular functions found to be significantly over- (right side of zero) or under- (left of zero) represented by the differentially-expressed peptides in the data set. Filled red circles highlight significant hits (FDR<0.3) with the size related to the corresponding rank value.

## Supporting Figure 9:

Phosphopeptide level enzyme terms enrichment for 72h of Mst4 overexpression. Volcano plot showing log2 enrichment against significance of enrichment, highlighting enriched enzyme terms found to be significantly over- (right side of zero) or under- (left of zero) represented by the differentially-expressed peptides in the data set. Filled red circles highlight significant hits (FDR<0.3) with the size related to the corresponding rank value. Known Protein kinase C (PKC) phospho sites were highly significantly enriched upon Mst4 overexpression.

**Supplemental Table:**

Supplemental table showing overall results from interactome studies of Mst4 overexpressed in NRVCM and compared to LacZ and with or without hesperadin treatment.
